# Impact of hyperglycemia and treatment with metformin on ligature-induced bone loss, bone repair and expression of bone metabolism transcription factors

**DOI:** 10.1371/journal.pone.0237660

**Published:** 2020-08-25

**Authors:** Fernando Souza Malta, Roberto Puertas Garcia, Josuel Siqueira Azarias, Geysica Kauane Dos Reis Ribeiro, Tamires Szemereske Miranda, Jamil Awad Shibli, Marta Ferreira Bastos

**Affiliations:** 1 Department of Periodontology and Oral Implantology, Dental Research Division, Guarulhos University, São Paulo, Brazil; 2 Department of Post-Graduation in Aging Sciences, São Judas Tadeu University, São Paulo, Brazil; Charles P. Darby Children’ss Research Institute, 173 Ashley Avenue, Charleston, SC 29425, USA, UNITED STATES

## Abstract

This study evaluated the influence of type 2 diabetes mellitus on bone loss, bone repair and cytokine production in hyperglycemic rats, treated or not with metformin. The animals were distributed as follow: Non-Hyperglycemic (NH), Non Hyperglycemic with Ligature (NH-L), Treated Non Hyperglycemic (TNH), Treated Non Hyperglycemic with Ligature Treated (TNH-L), Hyperglycemic (H), Treated Hyperglycemic (TH), Hyperglycemic with Ligature (H-L), Treated Hyperglycemic with Ligature (TH-L). At 40^th^ day after induction of hyperglycemia, the groups NH-L, TNH-L, H-L, TH-L received a ligature to induce periodontitis. On the 69^th^, the TNH, TNH-L, TH, TH-L groups received metformin until the end of the study. Bone repair was evaluated at histometric and the expression levels of Sox9, RunX2 and Osterix. Analysis of the *ex-vivo* expression of TNF-α, IFN-γ, IL-12, IL-4, TGF-β, IL-10, IL-6 and IL-17 were also evaluated. Metformin partially reverse induced bone loss in NH and H animals. Lower OPG/RANKL, increased OCN and TRAP expression were observed in hyperglycemic animals, and treatment with metformin partially reversed hyperglycemia on the OPG/RANKL, OPN and TRAP expression in the periodontitis. The expression of SOX9 and RunX2 were also decreased by hyperglycemia and metformin treatment. Increased *ex vivo* levels of TNF-α, IL-6, IL-4, IL-10 and IL-17 was observed. Hyperglycemia promoted increased IL-10 levels compared to non-hyperglycemic ones. Treatment of NH with metformin was able to mediate increased levels of TNF-α, IL-10 and IL-17, whereas for H an increase of TNF-α and IL-17 was detected in the 24- or 48-hour after stimulation with LPS. Ligature was able to induce increased levels of TNF-α and IL-17 in both NH and H. This study revealed the negative impact of hyperglycemia and/or treatment with metformin in the bone repair via inhibition of transcription factors associated with osteoblastic differentiation.

## 1. Introduction

Diabetes mellitus is a metabolic disorder with multiple etiology that generates a chronic systemic state of hyperglycemia, with Type 2 being more common, affecting approximately 90% of diabetic individuals. Among the pathologies exacerbated by diabetes Type 2, the effect of chronic hyperglycemia on the periodontal environment has been characterized by the prevalence and severity of periodontitis. Therefore, periodontal disease has be ranked as the sixth classical complication of diabetes [1]. Various mechanisms have been proposed to explain the interactions between diabetes and periodontal disease. Among them, it has been proposed that the increase in severity of periodontal disease in the presence of diabetes was caused by the increase in expression of inflammatory mediators, osteoclastogenic factors, metalloproteinases and consequent exacerbation of the inflammatory response caused by the advanced glycation end products (AGEs) (glycosylation) [2–4].

Although clinical studies are capable of pointing out diabetes mellitus as a risk factor for periodontal disease, there are many confounding factors that make it difficult to determine the real immune-inflammatory factors that link diabetes mellitus to periodontal disease. Therefore, the development of animal models has become essential to enable improved understanding of the relations between periodontitis and diabetes mellitus type 2. Rodents have been the first choice among small-sized animals, because they are easily manipulated and are low cost relative to housing and diet. Nevertheless, there is a scarcity of studies in the that have used experimental models of diabetes type 2 and ligature-induced periodontitis. In general, studies have demonstrated that diabetic animals have a greater and more prolonged inflammatory response, higher level of bone loss and reduction in bone neoformation when compared with nondiabetic animals [5,6]. In 2007, Pontes Andersen et al. [7] investigated whether the inflammation associated with periodontitis could contribute to the prediabetic state and thus contribute to the progression of diabetes in diabetic and obese rats of the Zucker lineage. The authors demonstrated that 4 weeks after inserting the ligature, the presence of periodontitis promoted disturbances in glucose homeostasis of diabetic rats, thereby harming the pre-diabetic state. Whereas, a higher level of bone loss has also been observed in these animals, when compared with normoglycemic rats. In 2008, the effect of experimental ligature-induced periodontitis associated with a diet rich in fats [8] was evaluated in diabetic and obese rats of the Zucker lineage relative to insulin resistance. The results showed that ligature-induced periodontitis harmed the glucose homeostasis in diabetic rats fed on a diet rich in fats. More recently [9], a pre-clinical study observed an increase in inflammatory response and osteoclastogenesis in Goto-Kakizaki rats with ligature-induced periodontitis via TNF production. Experimental studies evaluating the impact of diabetes type 2 on alveolar bone repair and even on the osseointegration of implants are also scarce in the literature. Hasegawa et al. [10] demonstrated a lower osseointegration capacity in type 2 diabetic rats, and in a similar manner Wang et al. [11] demonstrated rats of the Goto-Kakizaki lineage had a larger quantity of fibrous tissue around implants installed in the femoral regions that the nondiabetic control group animals had.

Studies involving ligature-induced periodontitis in nongenetic models of diabetes type 2 are even scarcer. One study demonstrated that diabetes affected the expression of mediators related to the establishment and progression of periodontal disease, such as TNF-α, IL-1, IL-6, MMP-2, MMP-9 and RANKL [12].

Metformin is the first-choice medication for the treatment of diabetic type 2 individuals who do not yet have any associated disease such as cardiomyopathies, retinopathies or kidney failure. It is one of the most widely used antihyperglycemic medications, and recent studies have demonstrated that this drug inhibits the harmful effects of diabetes on bone tissue, reducing the risk of fractures in patients [13,14]. Recently, Liu et al. [15] demonstrated that metformin diminished the RANKL/OPG proportion, the number of cells marked with TRAP, and consequently the areas of bone resorption in periapical lesions. However, up to now, the effects of hyperglycemia either associated or not with treatment with metformin on alveolar bone metabolism has not been evaluated in situations of repair or relative to the induction of periodontitis. Therefore, the aim of this study was to evaluate, by means of histometric, histochemical, immunohistochemical and molecular analyses, the influence of diabetes mellitus type 2, either associated with treatment with metformin, or not, on ligature-induced bone loss, alveolar bone repaid post-extraction and production of cytokines ex vivo in rats.

## 2. Material and methods

### 2.1 Selection of animals

In total, 80 Wistar rats aged 45 days, weighing approximately 300 g, acquired from the animal facility of the University of São Paulo (USP) were used. Before the experiment started, the animals were acclimatized to the vivarium environment of the Guarulhos University for a period of five days. Throughout the experimental period, all the animals were kept in individual cages for better control of fructose ingestion. They were kept under the same environmental conditions and received food and water *ad libitum*. All the experimental groups remained in alternate periods of light and darkness (12/12 hours); This study was approved by the Ethics Committee on the Use of Animals for Experimentation of the Guarulhos University (CEUA-UnG) Protocol No. 004/13.

### 2.2 Experimental groups and study design

At the beginning of the study, the animals were divided into the following experimental Groups:

- Nonhyperglycemic without Periodontitis (NH, n = 10): rats that received water without fructose, were inoculated with citrate buffer and did not receive the ligature.
- Nonhyperglycemic without Periodontitis (NH-L, n = 10): rats that received water without fructose, were inoculated with citrate buffer and received the ligature.
- Nonhyperglycemic without Periodontitis (NHT, n = 10): rats that received water without fructose, were inoculated with citrate buffer, were treated with metformin and did not receive the ligature.
- Nonhyperglycemic without Periodontitis (NHT-L, n = 10): rats that received water without fructose, were inoculated with citrate buffer, treated with metformin and received the ligature.
- Hyperglycemic without Periodontitis (H n = 10): rats that received water with fructose during the first two weeks of the experimental period, were inoculated with streptozotocin (STZ), and did not receive the ligature.
- Hyperglycemic without Periodontitis (HT n = 10): rats that received water with fructose, were inoculated with streptozotocin (STZ), treated with metformin and did not receive the ligature.
- Hyperglycemic with Periodontitis (H-L n = 10): rats that received water with fructose, were inoculated with streptozotocin (STZ), and received the ligature.
- Hyperglycemic with Periodontitis Treated (HT-L n = 10): rats that received water with fructose, were inoculated with streptozotocin (STZ), treated daily with metformin and received the ligature.

The experimental period consisted of a total of 84 days (S1 Fig), during which the animals received water only (NH, NHT, NH-L, NHT-L) or water with the addition of fructose (H, HT, H-L, HT-L) as from day zero. The animals belonging to Groups H, HT, H-L, HT-L were inoculated with streptozotocin (STZ), on the 14th day of the experimental period, while those belonging to Groups NH, NHT, NH-L, NHT-L were inoculated with citrate buffer on the same day. On a weekly basis, the groups were followed-up for analysis of the blood glucose levels by means of collection from the caudal vein. On the 54th day after the experiment began, and 40th day after induction of diabetes, Groups NH-L, NHT-L, H-L, HT-L received the ligature that remained in place for 30 days. On the 69th day after the experiment began, 55th day after induction of diabetes and 15th day after ligature placement, Groups NHT, NHT-L, HT, HT-L began daily treatment with metformin (40mg/Kg body weight) administered orally up to the end of the experimental period. On the 76th day all the animals were submitted to tooth extraction, which occurred eight days before the end of the experimental period.

### 2.3 Hyperglycemia induction

For the induction of hyperglycemia, the animals received water supplemented with 10% fructose *ad libitum* during the first two weeks of the experimental period, a total of 14 weeks. Afterwards, all the animals that consumed water supplemented with fructose were inoculated by intraperitoneal via with streptozotocin (STZ, 40 mg/Kg body weight) in Citrate buffer (pH 4.4). The animals belonging to the nondiabetic groups were inoculated with the vehicle only.

One week after STZ inoculation, the blood glucose levels were monitored with the aid of a portable blood glucose meter, by means of collections from the caudal vein. The animals belonging to Groups H, HT, H-L, HT-L in nonfasting situation that presented with levels > 300mg/dl were considered diabetic and remained in the study, while those that had levels lower than that established were removed from the study [16].

### 2.4 Ligature placement

At 54 days after the experimental period began, and 40 days after the induction of hyperglicemia, the animals belonging to Groups H-L, HT-L, H-L, HT-L received a ligature to induce an accumulation of biofilm and bone loss. All the animals were weighed and anesthetized via intraperitoneal injection with a solution of 10mg/Kg ketamine hydrochloride (Francotar^®^; Virbac do Brasil Indústria e Comércio LTDA, Roseira, SP, Brazil) and 0.1ml/kg xylazine hydrochloride (Virbaxil^®^; Virbac do Brasil Indústria e Comércio LTDA, Roseira, SP, Brazil). The animals belonging to Groups NH-L, NHT-L, H-L, HT-L received a threat of cotton around the right first molar, which was kept in place for 30 days.

### 2.5 Extraction

Eight days before euthanasia, (76th day of the experimental period) all the animals were anesthetized according to the previously described protocol. The first molars were extracted with the aid of a micro-chisel. The recently extracted alveoli localized on the right were used for histometric analyses, while those localized on the left were submitted to curettage and evaluated relative to gene expression of Sox 9, RunX 2 and Osterix, which are essential transcription factors for differentiation of mesenchymal cells into osteoblasts and consequently related to bone formation [17,18]. During the postoperative period, the animals received antibiotic therapy with pentabiotic (1 ml/kg, Wyeth-Whitehall Ltda) in a single dose via intramuscular injection and analgesia with tramadol hydrochloride (10 mg/kg), every 12 hours for a period of 48 hours.

### 2.6 Metformin administration

To evaluate the effects of metformin on bone tissue in the absence or presence of diabetes mellitus and periodontal disease, Groups NHT, NHT-L, HT, HT-L were treated with metformin at the dose of 40mg/kg diluted in mineral water via oral administration, as previously described by [15]. Treatment was performed from the 69^th^ to 84^th^ day of the experimental period, totaling 15 days.

### 2.7 Preparation of histological sections

Eighty-four days after the study began, the animals would be euthanized by means of an overdose of anesthesia by association of sodium pentobarbital (100 mg/kg) and Lidocaine (10mg/ml) via intraperitoneal injection.

The mandibles were removed and sectioned into blocks containing the right and left molars. In the same way, the maxillae were removed and sectioned in the proximities of the right first molar. The specimens were fixed in a solution containing 10% buffered formaldehyde solution at 4°C for 24h. After the fixation process and after washing in phosphate buffered saline solution (PBS) (pH7.4) at 4°C for 24 hours, the specimens were decalcified in 4.13% EDTA in Tris-HCl buffer, pH 7.4 at 4°C for a period of approximately 70 days. After this the specimens were dehydrated in a series of increasing grades of ethyl alcohol solution (60 to 100%) under constant agitation. The parts were then diaphanized in xylol, infiltrated with paraffin and embedded in paraffin blocks. From the blocks of the mandible, serial sections approximately 5 μm thick were obtained, starting from the cut where the vestibular root fails to appear. The mandibular first molars were sectioned in the vestibular-lingual direction to exhibit the inter-radicular area (furcation). The maxillary alveoli were sectioned in the transverse direction. Some of the sections were stained with hematoxylin and eosin, and other were destined for histochemical (TRAP) and histochemical analyses (OPG, RANKL, Osteocalcin and Osteopontin).

### 2.8 Gene expression analysis

#### 2.8.1. RNA extraction

After euthanasia, the alveoli were subjected to curettage and the granulation tissue was removed and stored in RNAlater^®^ solution (Ambion Inc.), to prevent degradation of the RNA. The samples were incubated at 4°C for a period of 24 hours and afterwards stored at -20°C until the time of extraction. Extraction was performed using TRIZOL^®^ reagent (Gibco BRL, Life Technologies, Rockville, MD, USA) in accordance with the manufacturer’s recommendations. The RNA samples were subsequently resuspended in approximately 20 μL of water treated with Diethyl pyrocarbonate (DEPC) and stored at –70°C. Total RNA quality was evaluated by means of 1% agarose gel electrophoresis.

#### 2.8.2 Treatment with DNAse and reverse transcription

The total RNA samples were treated to eliminate any residue of DNA with DNAse (DNA-free^™^, Ambion Inc.), as recommended by the manufacturer. Total RNA was quantified by means of a spectrophotometer and approximately 1 μg of the (DNA-free) total RNA sample was used for cDNA synthesis. For this purpose, the First-*strand cDNA synthesis* kit (Roche Diagnostic Co.), was used in accordance with the manufacturer’s recommendations. Initially, the samples were incubated for at 25°C for 10 minutes, and afterwards at 42°C for 60 minutes. On conclusion of the second stage of incubation, the samples were incubated at 95°C for 5 minutes, and afterwards at 4°C for 5 minutes to cool. The reagents used and their respective concentrations were buffer solution (1x), MgCl_2_ (5mM), deoxyribonucleotides (1mM), randomized *primers* (3.2μg), RNAse inhibitor (50U), and reverse trancriptase AMV (20U).

#### 2.8.3 Gene expression analysis by real-time PCR (RT-PCR)

*2*.*8*.*3*.*1 Design of primers*. The *primers* for GAPDH (reference gene), Sox9, RunX2 and Osterix were designed with the aid of a program developed specifically for the elaboration of primers for the *LightCycler* (Roche Diagnostics GmbH, Germany). All the primers were verified relative to their specificity by means of analyzing the Melting curve, at all times using a positive and negative control.

*2*.*8*.*3*.*2 RT-PCR reactions*. The RT-PCR reactions were performed with the *LightCycler* system (Roche Diagnostics GmbH, Mannheim, Germany), using the *FastStart DNA Master SYBR Green I* kit (Roche Diagnostics GmbH, Mannheim, Germany). The profile of reactions was determined in accordance with the protocol suggested by the equipment manufacturer. For each of the analyses, water was used as negative control, and the product of the reactions was quantified by using the program manufacturer’s own program (LightCycler Relative Quantification Software—Roche Diagnostics GmbH). The levels of the GAPDH gene expression were used as reference for normalization of the values (Table 1).

**Table 1:** Target gene, sequence of *primers*, amplification profile used and size of *amplicons* generated during the *Real Time* PCR reaction.

Gene	Sequence(5’-3’)	Aplification profile [temperature (°C)/time (s)]	Size of amplicons (bp)
Sox-9.	F:CCTGAAATCTGAGAGCCTG R:CCTGAAATCTGAGAGCCTG	95/10; 55/7; 27/7	213
Runx2	F:CTCCCCAGTTGTGATCTGG R:GAATTGTTGTGGGAAACAGGTG	95/10; 56/7; 72/7	156
Osterix	F:CCAGCTGCCTACTTACCC R:GTTTGCCTGCACCACTC	95/10; 56/7; 72/7	164
GAPDH	F:CTGAGTACGTCGTGGAGTC. R:TGATGATCTTGAGGCTGTTGTC	95/10; 56/5; 72/10	250

GADPH = Glycerin-Aldehyde-3-Phosphate-Dehydrogenase.

### 2.9 Histometric analyses

Histological sections stained with HE were digitized by means of NIS Elements software (Nikon). Interradicular bone loss of the right and left mandibular molars was analyzed in 10 previously selected, central, equidistant images (16 μm) per specimen. The area of newly formed bone within the alveolus was also obtained. Interradicular bone loss and the quantity of newly formed bone surrounding the region of interest values were calculated with the aid of a tool of the NIS Elements program itself. All analyses were performed by a single, blinded for experimental group and previously trained examiner.

### 2.10 Histochemical analyses for Tartrate-Resistant Acid Phosphatase (TRAP)

The histological sections of the nonhyperglycemic animals were incubated for at 37°C for 30 minutes in a humid chamber with a solution for the detection of Tartrate-Resistant Acid Phosphatase containing Naftol AS-BI phosphate (Sigma, St. Louis, USA), sodium acetate, *Fast Red Garnet GBC* (Sigma, St. Louis, USA), sodium tartrate and nitrate solution. After incubation, the sections were washed in running water for 30 minutes and counter stained with *Fast green* (Sigma, St. Paul, USA) for 50 seconds. The cells marked with TRAP were counted with the aid of an image analysis program (*NIS Elements*, Nikon) in the interradicular region (mandibular first molar).

### 2.11 Immunohistochemical analyses

Images were captured at 200× magnification. Immunohistochemical analyses were performed for evaluating osteocalcin (OCN), osteopontin (OPN), receptor activator of NF-КB ligand (RANKL) and osteoprotegerin (OPG) in bone tissues. Sections were mounted on glass slides pre-treated with 3-aminopropyltriethoxy-silane. Sections were treated with 3% hydrogen peroxide for 30 minutes to eliminate endogenous peroxidase and blocked with PBS-1% bovine serum albumin for 30 minutes before incubation with the primary antibody (GeneTex, Inc; polyclonal antibodies against OPN [1:200], OCN [1:200], RANKL [1:100] and OPG [1:100]) for 3 hours at room temperature. Subsequently, the sections were incubated with biotinylated secondary antibody for 45 minutes and were treated with the streptavidin peroxidase conjugate (GeneTex, Inc) for 30 minutes. The specific reaction for each antibody was visualized using 3,3′diaminobenzidine. Sections were counter-stained with Mayer’ss hematoxylin, dehydrated through graded ethanol, cleared in xylene, and mounted on slides with the aid of Permount mounting media (Permount mounting media, Thermo Fisher Scientific). Negative controls were obtained by omission of the primary antibodies. The intensity of the staining was scored using a semi-quantitative analysis in the bone crestal region of the furcation area and in the post-extraction alveoli using image analysis software (Image J, National Institute of Mental Health). A standardized checkered diagram was overlaid in these areas, forming a series of squares. Subsequently, the intensity of the staining dominant in each square was classified into no staining (0), weak staining (1), moderate staining (2), and strong staining (3), as previously described [19]. The final classification was obtained by the intensity of the staining that predominated in a given image (ie, greater number of squares classified with a given intensity). Furthermore, the numbers of stained cells for OCN, OPN, OPG, and RANKL (brown or brownish stained cells) were counted in a previously delimitated area in the interradicular area (mandibular first molars) and in the post-extraction alveoli, marked with the respective antibody were counted, using image analysis software (Image J, National Institute of Mental Health). Data were expressed as number of stained cells/mm^2^ of bone.

### 2.12 Spleen cell cultures for *ex vivo* cytokine induction

At the time of euthanasia, the spleen of animals belonging to all of the experimental groups (n = 5/group) was removed and macerated in RPMI 1640 medium (Gibco) with the aid of a nylon sieve to obtain the spleen cells. The cell suspension was transferred to plastic tubes with lids and centrifuged at 1500 rpm for 10 minutes. After this the cell sediment was resuspended in RPMI medium with the addition of 10% Fetal Bovine Serum and 40μg gentamycin, and the cell concentration was adjusted to 1 x 10^6^ cells/mL. One part of the cells placed in culture was stimulated with LPS of *Escherichia coli* (Sigma) and remained in culture at 37°C and 5%CO_2_ for 48 hours. Subsequently, the supernatant was collected and stored in plastic tubes with lids at -20°C until the time of quantifying the cytokines.

### 2.13 Quantification of cytokines

The levels of TNF-α, IFN-γ, IL-12, IL-4, TGF-β, IL-10, IL-6 and IL-17 were evaluated by ELISA. Briefly, 100μL of the samples were placed in culture plates previously sensitized with antibodies specifically for each of the cytokines, and then incubated. After initial washing the biotinylated antibody was added, and the samples continued to incubate for an undetermined period of time. Subsequently they were washed and incubated with streptavidin-phosphatase conjugate for 60 minutes, followed by further washing and incubation with substrate. After the period of 30 minutes, 50 μL of sulphuric acid was added to the wells to paralyze the reaction. The absorbance values for each sample were obtained by readout in a spectrophotometer at 450 nm. A standard curve was prepared from the absorbance values obtained for the standards provided by the manufacturer to obtain an equation that allowed the quantity of each cytokine to be calculated.

### 2.14 Statistical analysis

The values obtained for each animal arising from the histometric, histochemical, immunohistochemical, molecular and immunological analyses were used for calculating the means, with a final result being obtained for each independent group. All the results were submitted to the Shapiro-Wilk test for analysis of normality, which indicated the use of nonparametric methods. The hypothesis that diabetes type 2 and treatment with metformin would have an influence on the interradicular bone loss of mandibular molars with and without ligatures, on post-extraction bone repair, and expression of OPG, RANKL, TRAP, Osteopontin, Osteocalcin, and on gene expression of Sox9, Runx2 and Osterix and on the systemic level of cytokines was tested by the Kruskal Wallis test (Dunn test) with the level of significance established at 5%.

## 3. Results

### 3.1 Animals

It was possible to observe that all the animals gained weight during the study (p<0.05) and no significant difference was observed relative to the initial weight of the animals belonging to NH and H groups. However, on conclusion of the experimental period and consequently after the induction of diabetes, it was possible to observe that the animals of NH group showed a significantly higher weight than that of the animals in H group, as illustrated in S2 Fig.

**Fig 1:**
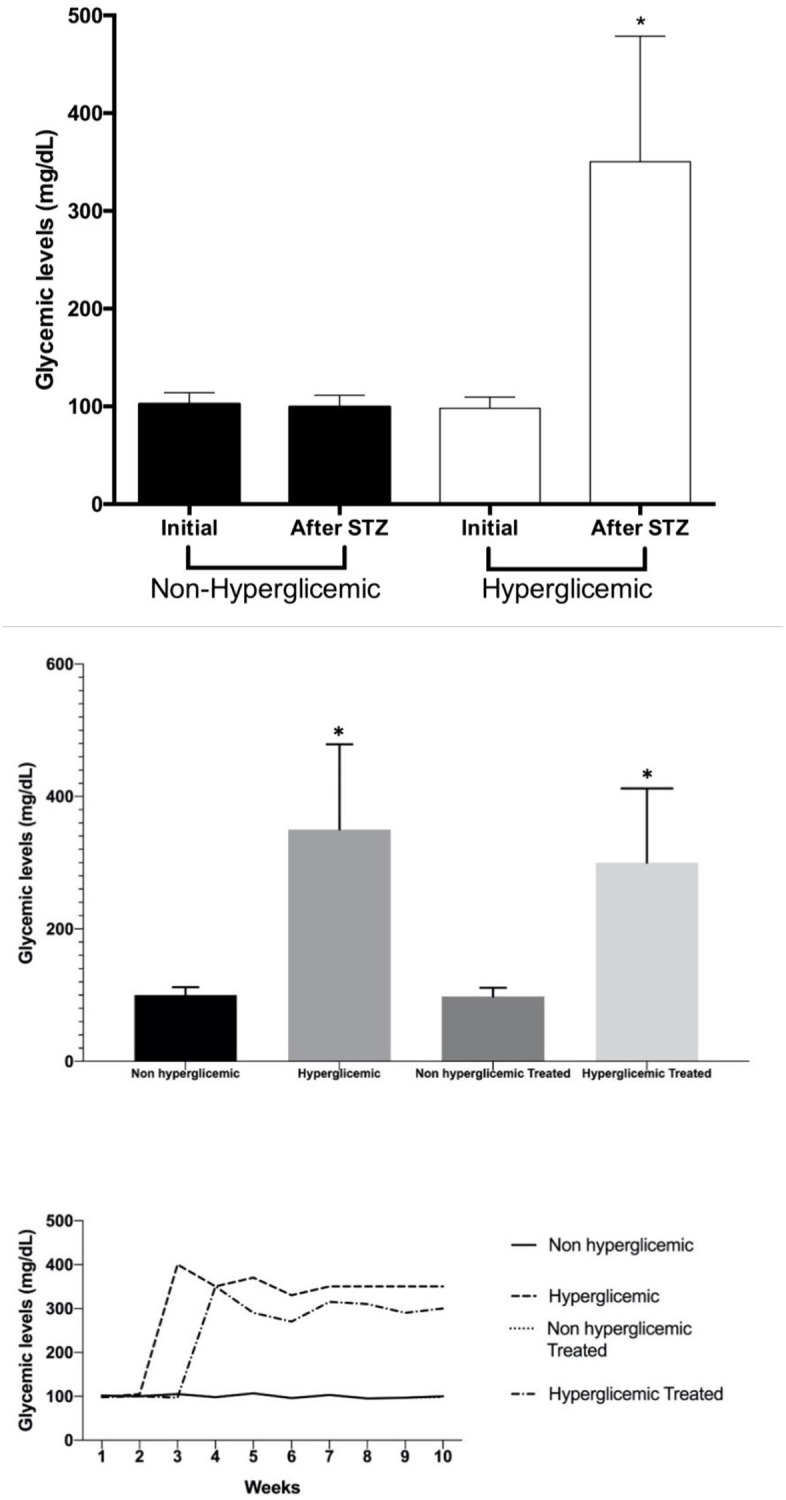
**A)** Initial and final glycemic levels of animals belonging to nonhyperglycemic (NH) and hyperglycemic (H) Groups. Dark bars represent the animals of Group NH, and white bars the animals of Group H. No significant differences were detected for initial and final glycemia for Group NH (Student’s-*t* test for dependent samples). Symbol represent significant difference among the groups relative to initial and final glycemic levels, separately evaluated by the Student’s-*t* test for independent samples; **B)** Mean and SD of Glycemic Levels of animals of all groups. Symbol represent significant difference among the groups, separately evaluated by the Student’s-*t* test for independent samples; **C)** Glycemic levels (mg/dL) for each group for 10 weeks.

The glycemic levels in nonfasting condition of the animals belonging to NH and H groups were monitored on a weekly basis, and no significant difference was observed among the groups for this parameter in the beginning of the experimental period (p>0.05). The mean and standard deviation for NH was 102 ± 12 mg/dl and for H it was 98 ± 11 mg/dl. After 14 days of fructose ingestion, the animals belonging to H group received STZ via interperitoneally injection, while the animals of NH group received only the citrate buffer (vehicle) by the same pathway. The glycemic levels were measured again 72 hours after this stage of the experiment. A significant increase in glycemia was observed for the animals of H in comparison with NH group (p<0.05). The mean and standard deviation for Group NH was 100 ± 12 mg/dl and for Group H it was 350 ± 129 mg/dl, as shown in Fig 1.

Treatment with metformin for 15 days was incapable of affecting the glycemic levels of the animals of NH and H groups when compared with NHT and HT groups (p = 0.05). The mean and standard deviation for NHT and HT groups was 98 ± 13 mg/dL and 300 ± 112 mg/dl, respectively.

### 3.2 Impact of hyperglycemia and treatment with metformin on ligature-induced alveolar bone loss

The histological sections of the mandibles stained with Hematoxylin and Eosin were used to perform the histometric analyses of alveolar bone loss in hyperglycemic and nonhyperglycemic animals with and without ligatures, either treated or not with metformin. The results were illustrated in Fig 5.

**Fig 2:**
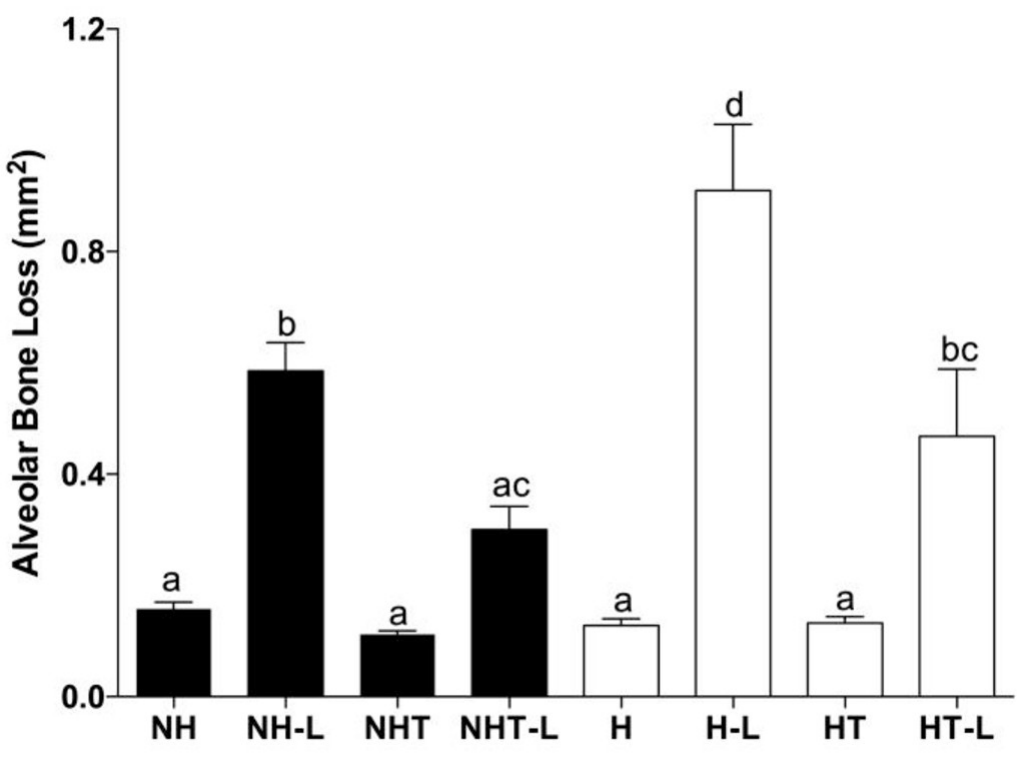
Alveolar bone loss in the furcation area of the mandibular first molar of nonhyperglycemic (NH) and hyperglycemic (H) rats treated (T) or not with metformin and in the presence or absence of ligature (L). Different letters represent significant differences among the groups evaluated by the Kruskall-Wallis nonparametric test (p<0.05).

Initially, no significant differences in alveolar bone loss were detected for the animals without ligature belonging to the different experimental groups (p>0.05). However, the ligature was capable of promoting a significant increase in alveolar bone loss when compared with that of animals without ligature (p>0.05). This result confirmed the efficacy of ligature for inducing the process of experimental periodontitis. Interestingly, the results observed for NHT-L group suggested that the ligature-induced bone loss process in nonhyperglycemic animals was attenuated by the treatment with metformin (p>0.05; Fig 2).

For the animals belonging to the groups with ligature-induced periodontitis, it was possible to note that the presence of hyperglycemia promoted a higher level of alveolar bone loss when compared with that of nonhyperglycemic animals. Also interesting was that it was possible perceive that although treatment with metformin had not significantly reduced the glycemic levels, it was capable of diminishing bone loss in both hyperglycemic and nonhyperglycemic rats (Fig 2).

#### 3.2.1 Impact of diabetes and treatment with metformin on immunohistochemical analyses of OPG, RANKL, OCN and OPN in mandibles of animals with or without ligature-induced periodontitis

**Fig 3:**
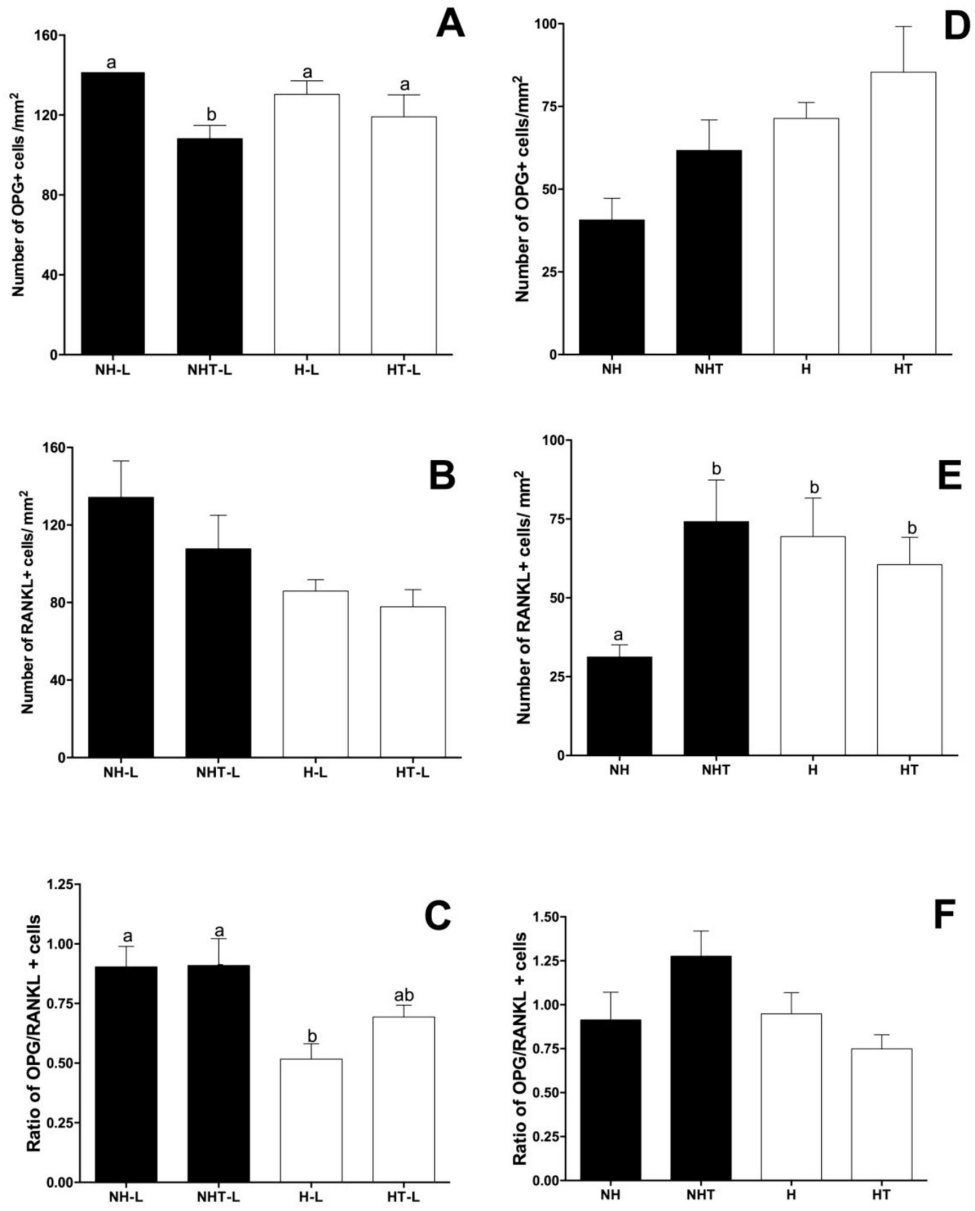
Mean and standard deviation of the counts of the number of cells positive for OPG (A and D) and RANKL (B and E) and of the OPG/RANKL proportion (C and F) in the region of the furcation area for the Nonhyperglycemic (NH), Nonhyperglycemic Treated (NHT), Hyperglycemic (H) and Hyperglycemic Treated (HT).

The number of OPG and RANKL-producing cells was obtained by immunohistochemical analyses, and the proportion of OPG/RANKL positive cells in NH and H animals, either treated or not with metformin, in the presence or absence of ligature. It was observed that the animals with ligature presented a lower number of OPG+ cells for the group of nonhyperglycemic animals treated with metformin in comparison with the other groups (Fig 3A). Although no differences were observed in the number of RANKL+ cells (Fig 3B), the proportion of OPG/RANKL was found to be diminished in the hyperglycemic animals (Fig 3C). Interestingly, the treatment with metformin for 15 days partially reverted this deleterious effect of hyperglycemia on the proportion of OPG/RANKL in hyperglycemic rats (Fig 3D).

In the absence of ligature, a higher number of RANKL+ cells were observed in the hyperglycemic animals when compared with the nonhyperglycemic rats. Furthermore, it was possible to note that the treatment with metformin promoted an increase in the number of RANKL+ cells in nonhyperglycemic animals (Fig 3E). However, due to the absence of difference relative to the number of cells positive for OPG between the groups (Fig 3D), no changes were detected in the OPG/RANKL proportion for the different experimental groups of this study (Fig 3F).

**Fig 4:**
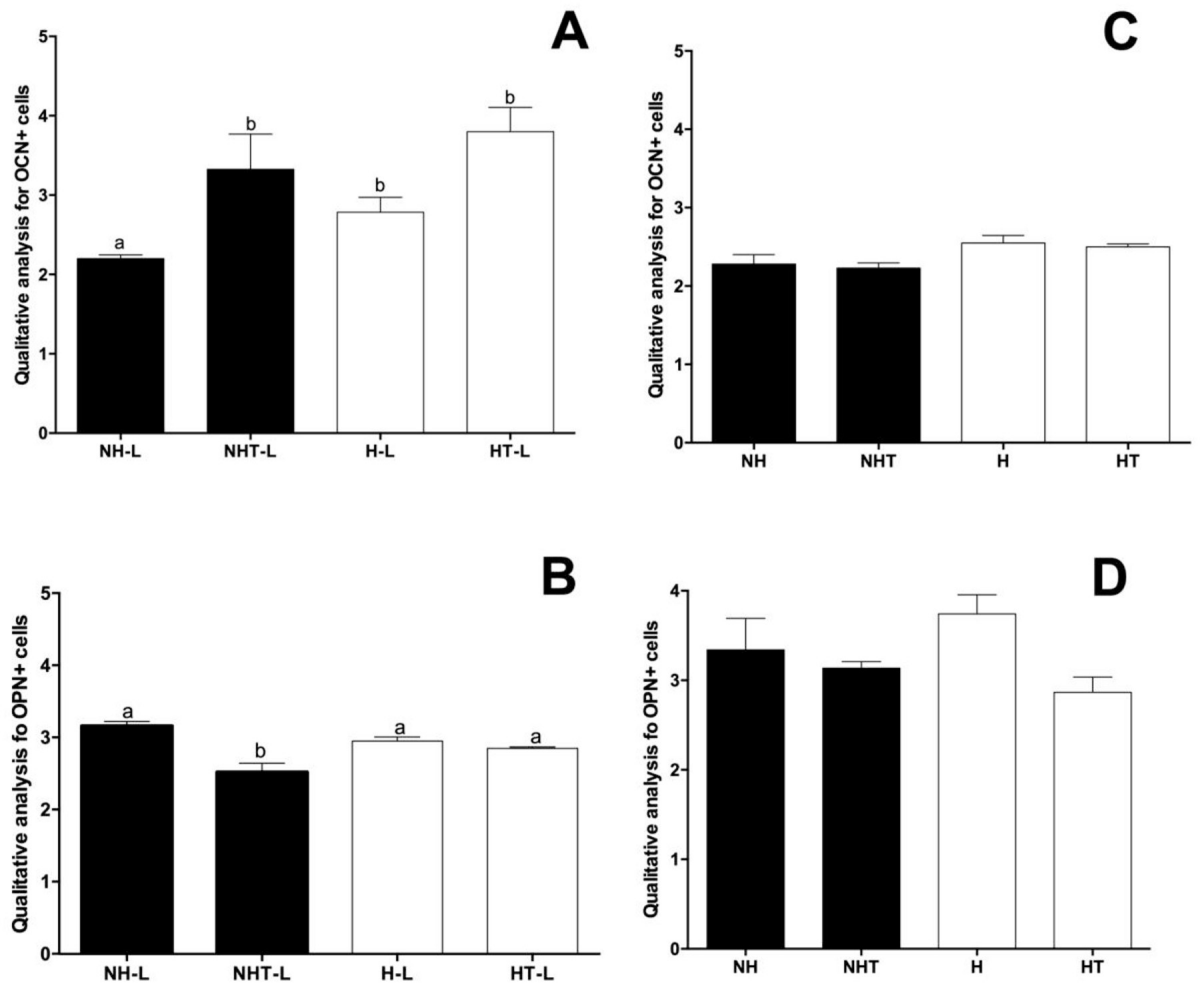
Mean and standard deviation of the quantitative analysis of expression of cells positive for Osteocalcin (OCN, A and C) and Osteopontin (OPN, B and D) in the furcation area of the mandibular first molar of animals belonging to the Nonhyperglycemic (NH) and Hyperglcycemic (H) Groups, treated (T) or not with metformin, in the presence (A and B) or absence (C and D) of ligature (L) and Hyperglycemic Treated Groups. Different letters represent significant differences among the groups evaluated by the Kruskall-Wallis nonparametric test (p<0.05).

The immunohistochemical analyses showed no differences relative to the amount of OCN expression in the furcation area in the mandible of animals belonging to the different experimental groups, in the absence of ligature. However, in the presence of ligature, an increase in OCN expression could be noted for both hyperglycemic and nonhyperglycemic animals (Fig 4A). Similar results were observed in the analysis of OPN expression, in which no difference was detected relative to the quantity of marking of OPN expression in the furca region in the mandible of animals belonging to the different experimental groups, in the absence of ligature (Fig 4C). In the presence of ligature, a reduction in OPN expression could be noted for the nonhyperglycemic animals treated with metformin. No differences were detected for the hyperglycemic animals either treated or not with metformin (Fig 4B).

#### 3.2.2 Impact of diabetes and treatment with metformin on histochemical analyses of TRAP in mandibles of animals with or without ligature-induced periodontitis

By means of immunohistochemical analyses, the number of OPG and TRAP-producing cells was obtained, and after this, the proportion of cells positive for OPG/RANKL in NH and H animals, either treated or not with metformin, in the presence or absence of ligature.

**Fig 5:**
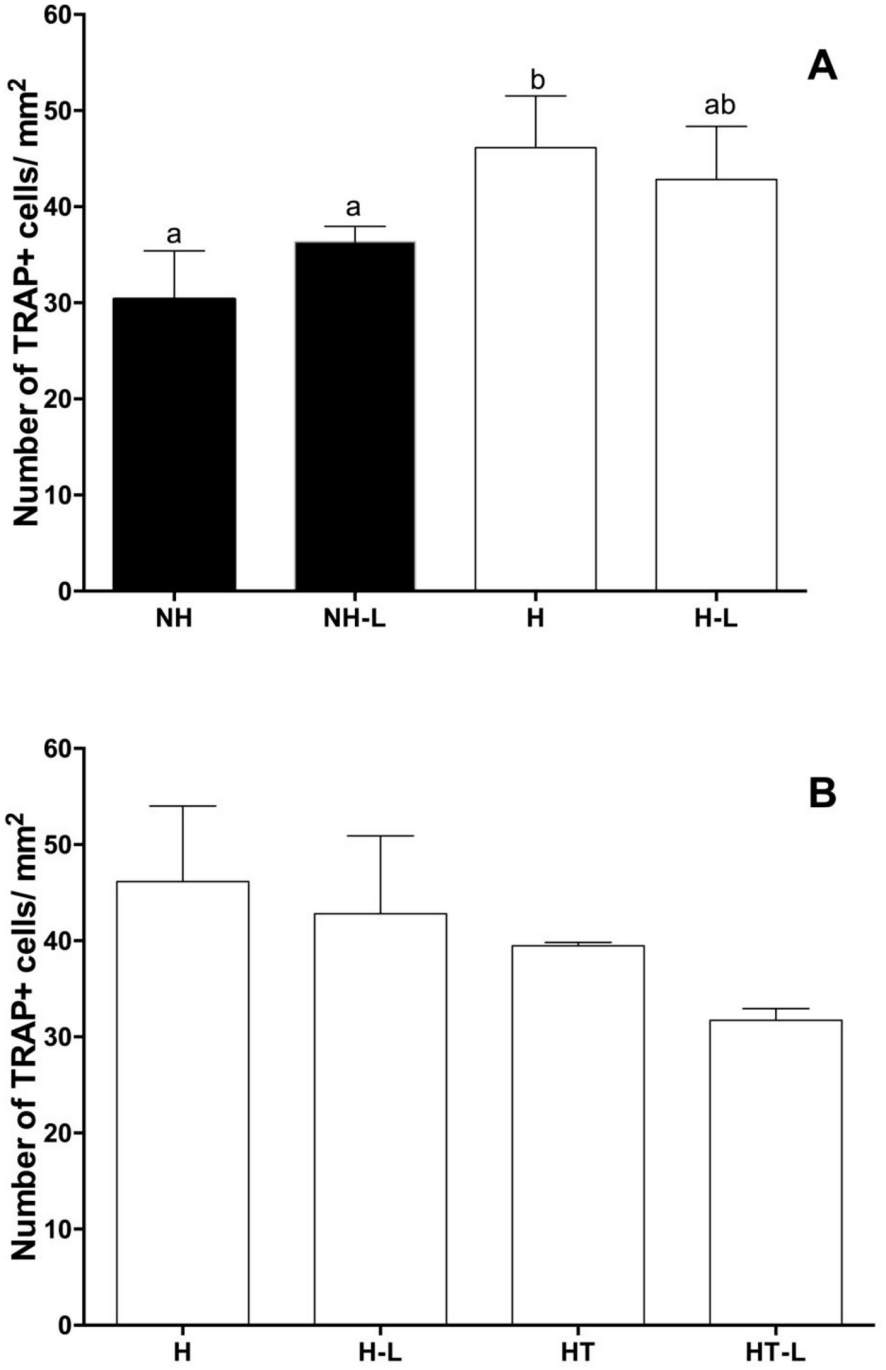
Mean and standard deviation of the quantitative analysis of expression of cells positive for TRAP+ cells in the furcation area of the mandibular first molar of Nonhyperglycemic (NH) in the absence and presence of ligature (L) (A) and Hyperglycemic (H) animals in the presence and absence of ligature, treated (T) or not with metformin (B), Different letters represent significant differences among the groups evaluated by the Kruskall-Wallis nonparametric test (p<0.05).

In the absence of treatment with metformin it was possible to note that hyperglycemia was capable of promoting an increase in the number of cells that expressed TRAP when compared with nonhyperglycemic rats (Fig 5A).

The hyperglycemic animals were evaluated with respect to the effect of treatment on the expression of TRAP producer cells. Although a trend towards the reduction in the number of TRAP producer cells could be observed in hyperglycemic animals treated in the presence of ligature, no significant differences were detected (Fig 5B).

### 3.3 Impact of diabetes and treatment with metformin on initial stages of alveolar bone repair

**Fig 6:**
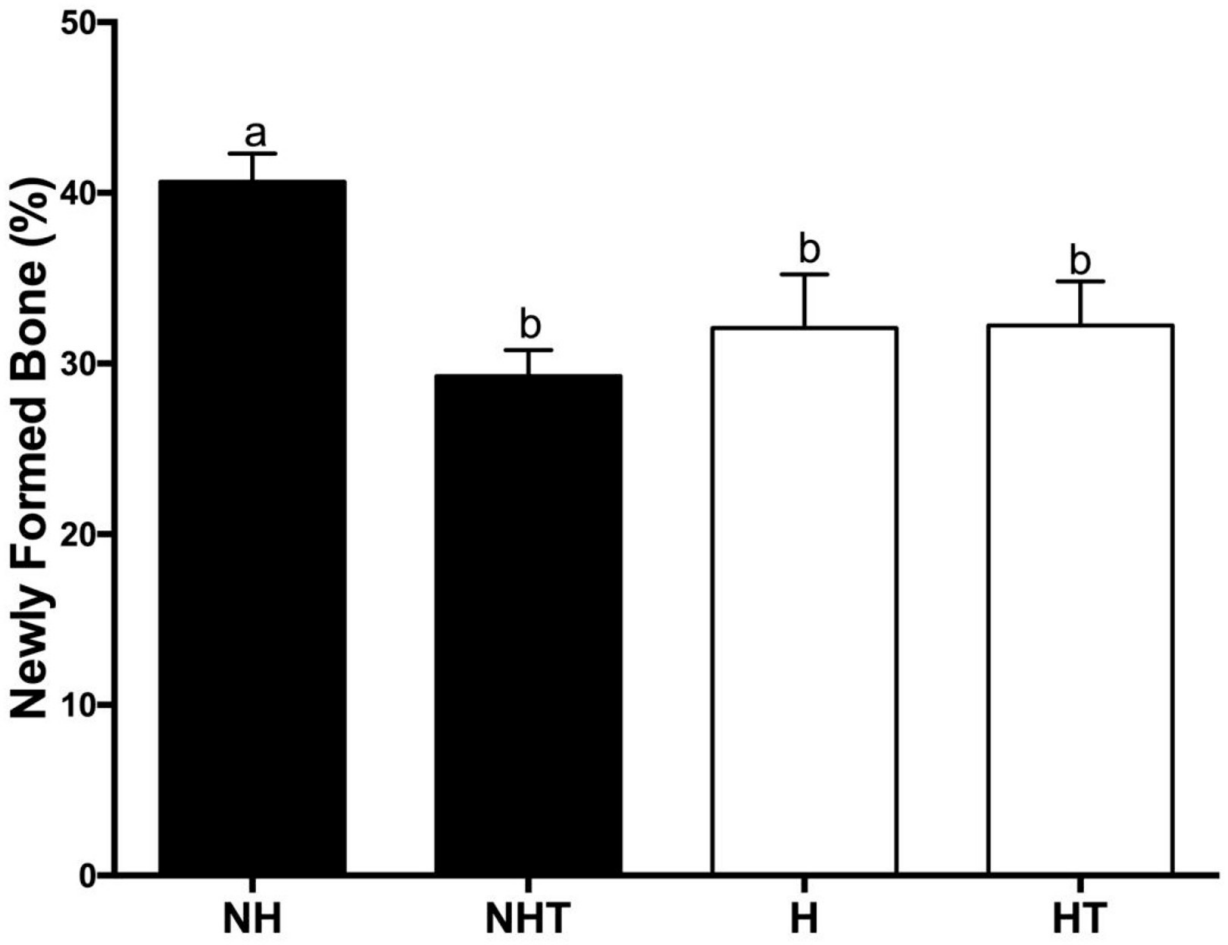
Percentage of newly formed bone in alveoli of rats 8 days post-extraction for different experimental groups. Different letters represent significant differences among the groups evaluated by the Kruskall-Wallis nonparametric test (p<0.05).

**Fig 7:**
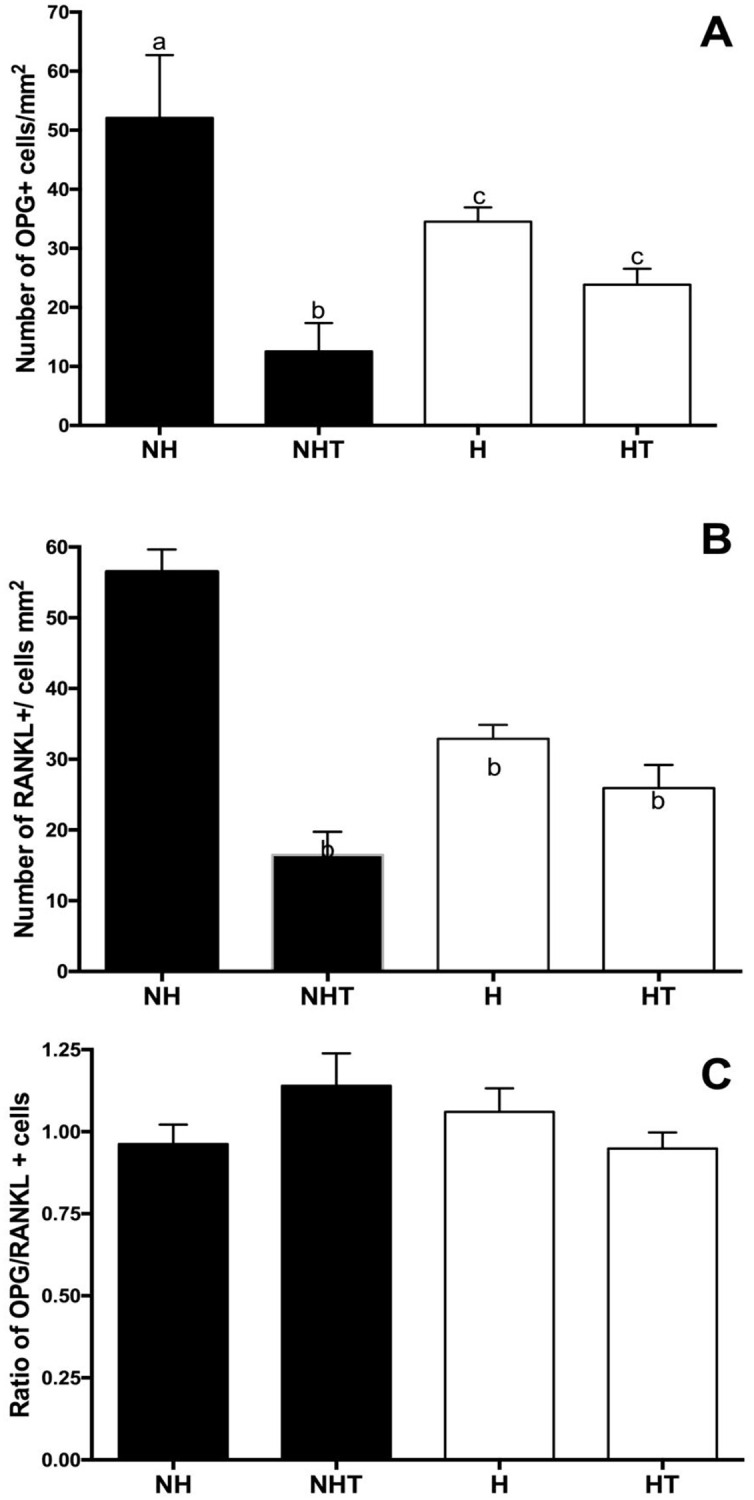
Mean and standard deviation of the counts of the number of cells positive for OPG (A) and RANKL (B) and of the OPG/RANKL proportion (C) in the alveoli of rats 8 days after extraction for the Nonhyperglycemic (NH), Nonhyperglycemic Treated (NHT), Hyperglycemic (H) and Hyperglycemic Treated (HT) Groups. Different letters represent significant differences among the groups evaluated by the Kruskall-Wallis nonparametric test (p<0.05).

The histological sections stained with Hematoxylin and Eosin were used for performing the histometric analyses of alveolar bone repair 8 days after extraction and the results were illustrated in Fig 6 and S4–S6 Figs. Significantly lower levels of bone neoformation were observed in the alveoli of hyperglycemic animals when compared with those of the nonhyperglycemic group of animals (Fig 7). The present study also evaluated the effect of treatment with metformin on the initial stages of alveolar bone repair in both normoglycemic and hyperglycemic animals. Unexpectedly, it was possible to observe that the treatment with metformin had a negative effect on bone neoformation of the normoglycemic animals. In the hyperglycemic animals’ metformin promoted no significant changes in alveolar bone repair. Illustrative images of the histological sections are presented in S3 Fig.

#### 3.3.1 Immunohistochemical analyses of alveolar bone repair

By means of immunohistochemical analyses, the numbers of OPG and RANKL producer cells were obtained. A lower number of OPG producer cells were detected in hyperglycemic animals either treated or not with metformin when compared with those of the nonhyperglycemic group. Normoglycemic animals treated with metformin also had a lower number of OPG producer cells when compared with untreated normoglycemic animals, and with the hyperglycemic groups of animals that were either treated or not (Fig 7A). In Fig 7B the results of the counts of RANKL producer cells are illustrated. It was possible to observe a lower number of cells expressing this marker in the hyperglycemic animals either treated or not with metformin and the treated normoglycemic rats, when compared with the nonhyperglycemic group. The result of the analysis of the OPG/RANKL proportion for the different experimental groups showed absence of significant differences between nonhyperglycemic and hyperglycemic animals (Fig 7C).

Representative images of the results of immunohistochemical analyses of OPG and RANKL may be found in S4A–S4D and S4E–S4G Fig, respectively.

**Fig 8:**
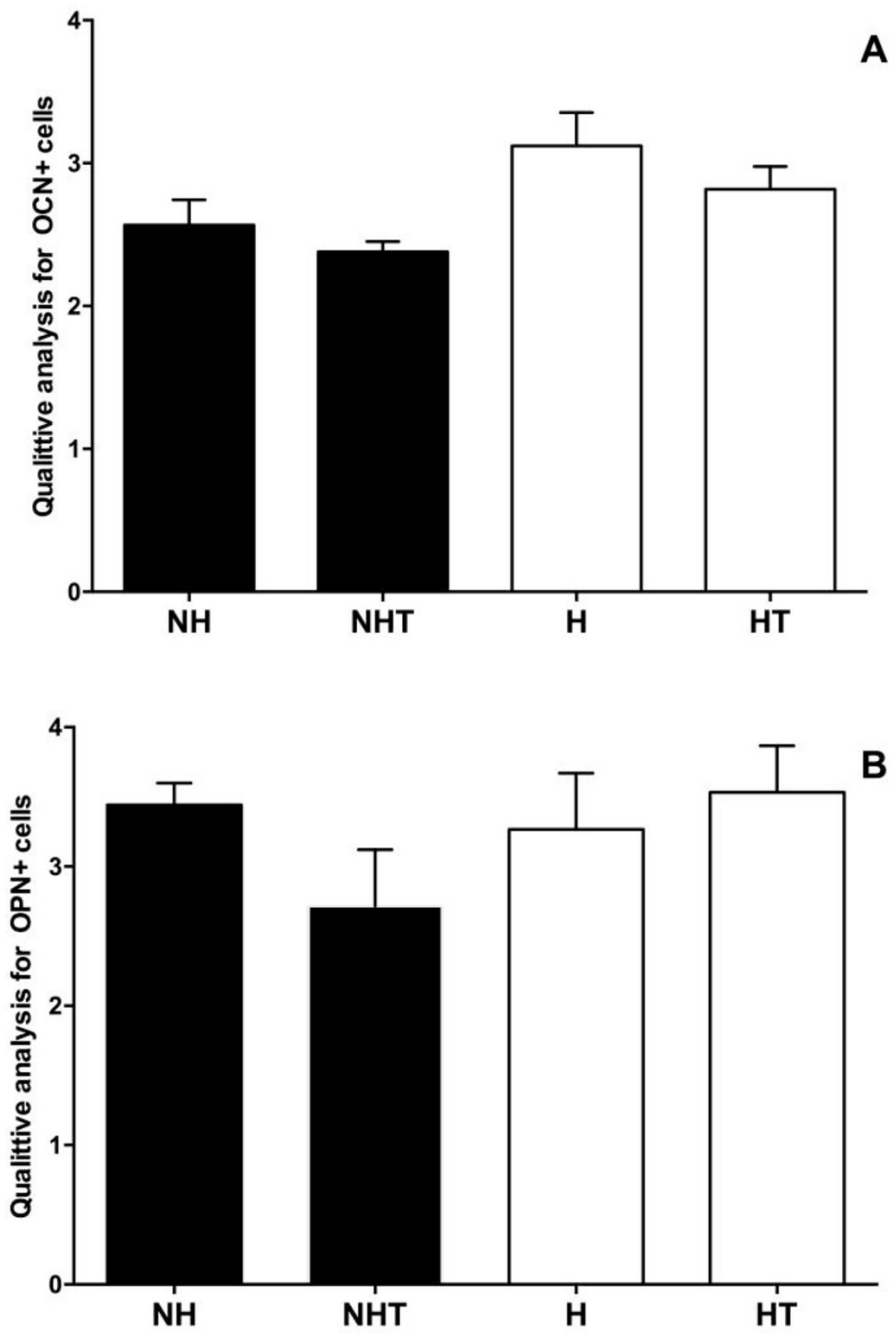
Mean and standard deviation of the quantitative analysis of expression of OCN (A) and OPN (B) in the alveoli of rats 8 days after extraction for the Nonhyperglycemic (NH), Nonhyperglycemic Treated (NHT), Hyperglycemic (H) and Hyperglycemic Treated (HT) Groups. Different letters represent significant differences among the groups evaluated by the Kruskall-Wallis nonparametric test (p<0.05).

Qualitative analyses of immunohistochemical reactions for OCN and OPN revealed no significant differences between normoglycemic and hyperglycemic animals treated or not with metformin in the different experimental groups. These results were illustrated in Fig 8 and S5–S6 Figs.

#### 3.3.2 Gene expression

The granulation tissue collected from the alveoli 8 days after extraction were submitted to the total RNA extraction process, treatment with DNAse and preparation of cDNA. After this, *Real time* PCR was performed for detection of the reference gene expression (GAPDH).

**Fig 9:**
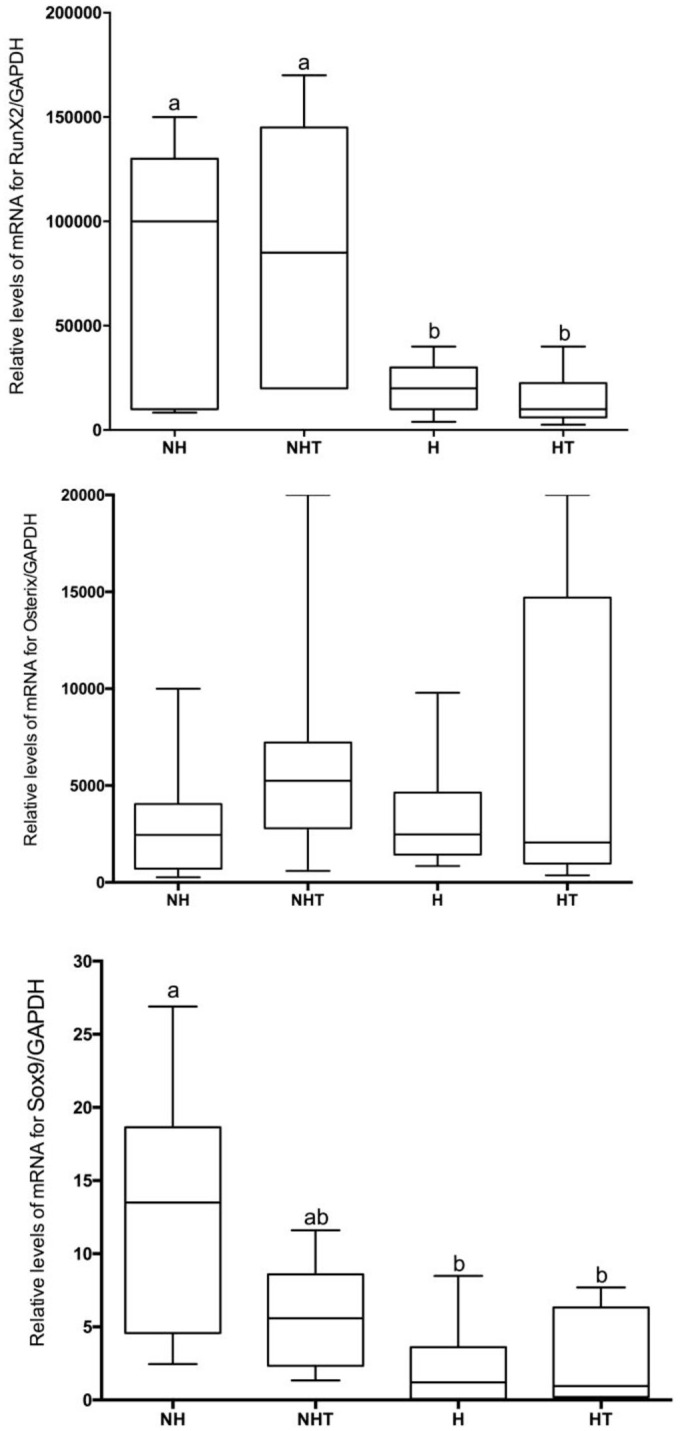
Levels of gene expression of RunX-2, Sox-9 and Osterix, in relation to reference gene (GAPDH = glycerin-aldehyde-3- phosphate-dehydrogenase) for different experimental groups. Horizontal bar represents median values. Different letters represent significant differences among groups evaluated by Kruskall-Wallis nonparametric test (p<0.05).

Relative to the transcription factors, it was possible to observe that the diabetic and diabetic treated animals had lower levels of gene expression of Runx2 and SOX9 when compared with nondiabetic animals (p<0.05). Furthermore, treatment with metformin was incapable of modifying the gene expression pattern of RunX2 and SOX-9 in both diabetic and normoglycemic animals. No significant differences in the levels of mRNA expression encoder for Osterix were observed for the different groups (Fig 9).

### 3.4 Impact of diabetes and treatment with metformin on *ex vivo* production of cytokines by spleen cells

The impact of hyperglycemia, ligature-induced bone loss and metformin on the expression of cytokines in the absence or presence of in vitro stimulation with LPS of rat spleen cells was evaluated by ELISA.

**Fig 10:**
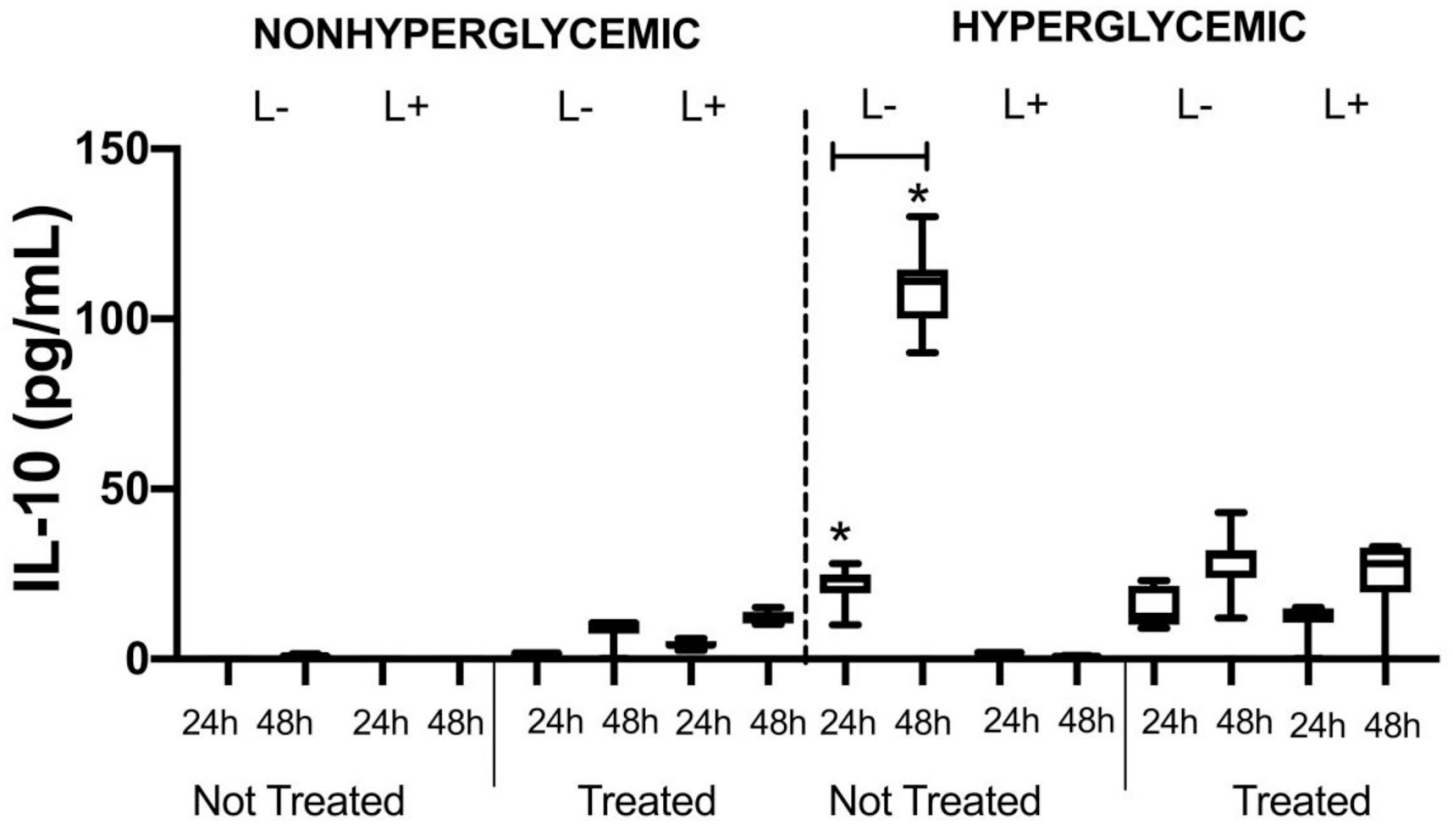
Median values of IL-10 levels (pg/mL) of supernatant of cultures of spleen cells collected from nonhyperglycemic and hyperglycemic animals, either treated or not with metformin in the absence (L-) or presence (L+) of ligature. * represent significant differences among the experimental groups for each period of supernatant collection evaluated by the nonparametric Kruskal Wallis test (p<0.05).

In the absence of *in vitro* stimulus, no presence of TNF-α, IL-6, IFN-γ, IL-4, IL-12, IL-17 and TGF-β was detected for any of the experimental Groups in the two-time intervals of evaluation of the supernatants (24 and 48 hours). The only cytokine with basal levels of production detected was IL-10. Nontreated hyperglycemic animals and without ligatures were observed to show higher levels of this cytokine when compared with normoglycemic (p<0.05) and hyperglycemic treated rats (p = 0.047). An increase in the levels of IL-10 was also observed in hyperglycemic animals that received the ligature when compared with hyperglycemic animals without the ligature (p = 0.027). The median values of the cytokine levels evaluated in the sample of the supernatant of spleen cell cultures not stimulated *in vitro* were presented in Fig 10.

In vitro stimulation of spleen cells with LPS promoted an increase in the levels of cytokines TNF-α, IL-6, IL-4, IL-17. However, cytokines IFN-γ, IL-12 and TGF-β showed no change in their levels after stimulation with LPS, as may be observed in Tables 1 and 2. It could also be noted that the levels of IFN-γ, IL-12 e TGF-β were not changed by hyperglycemia, treatment with metformin and certainly not by the absence and presence of ligature. This suggests that these factors had no influence on the systemic levels of these cytokines (Table 2).

**Table 2:** Median values of cytokine levels (pg/mL) of supernatant of cultures of spleen cells stimulated *in vitro* with LPS, collected from nonhyperglycemic and hyperglycemic animals, either treated or not with metformin in the absence or presence of ligature (n = 8/Group).

EXPERIMENTAL GROUPS	NONHYPERGLYCEMIC								HYPERGLYCEMIC							
	Not treated				Treated				Not treated				Treated			
	Without ligature		With ligature		Without ligature		With ligature		Without ligature		With ligature		Without ligature		With ligature	
Cytokines (pg/mL)	24h	48hs	24h	48hs	24h	48hs	24h	48hs	24h	48hs	24h	48hs	24h	48hs	24h	48hs
TNF-α	0.0(a)	0.0(a)	12(b)	14(a)	20(b)	22b	13b	20(b)	0.0(a)	0.0(a)	12(b)	16(a)	219(b)	28(b)	8.3(b)	26(b)
IL-6	0.0(a)	0.0(a)	4.8(a)	15(a)	0.0(a)	0.0(a)	0.0(a)	24(a)	0.0(a)	0.0(a)	0.0(a)	27(a)	9(a)	35(a)	30(b)	62(b)
IFN-γ	0.0(a)	0.0(a)	0.0(a)	0.0(a)	0.0(a)	0.0(a)	0.0(a)	0.0(a)	0.0(a)	0.0(a)	0.0(a)	0.0(a)	0.0(a)	0.0(a)	0.0(a)	0.0(a)
IL-4	0.0(a)	0.0(a)	0.0(a)	0.0(a)	0.0(a)	0.0(a)	0.0(a)	0.0(a)	31(a)	0.0(a)	160(b)	0.0(a)	289(b)	0.0(a)	419(b)	0.0(a)
IL-10	2.2(a)	22(a)	9.3(a)	0.0(a)	58(b)	125(b)	93(b)	165(b)	54(b)	260(b)	138(b)	114(b)	128(b)	322(b)	179(b)	196(b)
IL-12	0.0(a)	0.0(a)	0.0(a)	0.0(a)	0.0(a)	0.0(a)	0.0(a)	0.0(a)	0.0(a)	0.0(a)	0.0(a)	0.0(a)	0.0(a)	0.0(a)	0.0(a)	0.0(a)
IL-17	0.0(a)	0.0(a)	18(b)	72(b)	2.2(a)	68(b)	3.8(a)	96(b)	0.0(a)	23(a)	14(b)	111(b)	0.8(a)	80(b)	15(b)	192(b)
TGF-β	0.0(a)	0.0(a)	0.0(a)	0.0(a)	0.0(a)	0.0(a)	0.0(a)	6.2(a)	0.0(a)	0.0(a)	0.0(a)	0.0(a)	0.0(a)	0.0(a)	0.0(a)	0.0(a)

*Lower-case letters represent significant differences among the experimental groups for each period of supernatant collection evaluated by the nonparametric Kruskal Wallis test (p<0.05).

Proinflammatory cytokines (TNF-α and IL-6) were evaluated. It was possible to observe that when stimulated with LPS, the supernatant of the spleen culture cells showed a significant increase in the levels of TNF-α and IL-6 (p<0.001). Analysis of the impact of hyperglycemia on TNF-α synthesis revealed that the levels did not differ among the nonhyperglycemic and hyperglycemic groups 24 and 48 hours after *in vitro* stimulation. However, the ligature was capable of increasing the levels of TNF-α synthesis in both the nonhyperglycemic and hyperglycemic animals 24 hours after *in vitro* stimulation. Interestingly, the treatment with metformin promoted an increase in TNF-α synthesis in nonhyperglycemic animals in both the absence and presence of ligature after 24 and 48 hours of stimulation. Similar results were observed for the hyperglycemic animals that also showed significantly higher levels of TNF-α when compared with animals not treated with metformin after stimulation with LPS. For IL-6 differences were only observed in the treated hyperglycemic animals, which had higher levels of this cytokine when compared with those of the other groups.

The levels of cytokines considered anti-inflammatory, IL-4 and IL-10 were also increased after *in vitro* stimulus with LPS, and differences in the production profile of these cytokines were detected in the different experimental groups. For IL-4, hyperglycemic animals were observed to have increased levels of this cytokine in the first 24 hours after stimulation, in the Groups with ligature and treated with metformin. No changes were detected in the levels of IL-5 for the different groups of nonhyperglycemic animals in any of the periods evaluated; and for the hyperglycemic rats, 48 hours after stimulation (Table 2). For IL-10, nonhyperglycemic animals were observed to have lower levels of IL-10 when compared with those of hyperglycemic rats. Different from the data for hyperglycemic animals, it could be noted that nonhyperglycemic animals treated with metformin presented an increased level of this cytokine, both in the presence and absence of ligature in both periods of analysis.

Alterations in the levels of IL-17 were detected for the different experimental groups in this study. No effect of hyperglycemia was observed upon the levels of IL-17 in hyperglycemia, however, animals with ligature presented the higher levels of IL-17 when compared with nonhyperglycemic rats without ligature and hyperglycemic animals not treated with metformin. For the groups treated with metformin, this effect of the ligature was observed only in the period of 48 hours after LPS stimulation in the nonhyperglycemic and both periods for the Hyperglycemic rats (Table 2).

## 4 Discussion

For induction of type 2 diabetes, fructose administration and streptozotocin inoculation were used in the present study, as described by Islam and Wilson [20]. In this study, hyperglycemia was induced successfully, in agreement with earlier study that found an increase in glycemia after intraperitoneal administration of STZ [21]. However, no increase in ligature-induced bone loss was observed in hyperglycemic animals when compared with nonhyperglycemic rats. Different studies have demonstrated the increase in bone loss in hyperglycemic rats when compared with nonhyperglycemic animals, both in models of hyperglycemia induction with aloxan [22], streptozotocin [23–26] and in genetic models [27]. Therefore, these data suggested that the diabetes induction model using fructose and streptozotocin was not an adequate model for investigating the increase in ligature-induced alveolar bone loss.

Interestingly, the treatment with metformin was capable of partially reverting the ligature-induced bone loss both in nonhyperglycemic and hyperglycemic animals. Although metformin is the medication of choice for the treatment of type 2 diabetes, only two studies have evaluated the effects of this drug on alveolar bone loss in rats [15,28]. In agreement with the results obtained in the present study, other authors have also demonstrated that nonhyperglycemic rats treated with metformin showed lower bone loss values when compared with the untreated animals [15,28]. Although no changes were detected in the OPG/RANKL, OCN and OPN proportions for the different experimental groups in the absence of ligature-induced periodontitis, the present study also showed a lower OPG/RANKL proportion, and increase in expression of OCN and TRAP in the hyperglycemic animals, results that were in agreement with those described by Silva et al. [23] and Gennaro et al. [25]. In the present study it was also shown that treatment with metformin partially reverted the effect of hyperglycemia on the OPG/RANKL proportion, expression of OPN and TRAP in the presence of ligature-induced periodontitis. These results are in agreement with those obtained by Liu et al. [15]. The authors reported a reduction in the number of cells positive for RANKL and TRAP in animals that were treated with metformin, suggesting an osteo-protective effect of this medication.

In spite of different studies having described the lower repair potential in diabetic animals, few have evaluated the involvement of OPG and RANKL in bone repair associated with hyperglycemia. In the present study, a reduction in the levels of OPG and RANKL was observed in the hyperglycemic animals when compared with the normoglycemic rats. These results are in agreement with those obtained by de Amorim et al. [29], who used rats with hyperglycemia induced by aloxan, and performed immunohistochemical evaluation of the expression of OPG, RANK and RANKL and the RANKL/OPG proportion in the regions of bone repair at time intervals of 7 and 14 days after creating a bone defect in the tibias. The authors observed a reduction in the number of cells positive for OPG, RANK and RANKL in diabetic rats in 7 days. Although these data may suggest a negative impact of hyperglycemia on bone tissue, analysis of the RANKL/OPG or OPG/RANKL proportions more adequately represent the impact of hyperglycemia on the initial stages of alveolar bone repair in the animals, because these mediators present combined activities. In the present study, no significant differences were detected in the OPG/RANKL proportion between the hyper- and normoglycemic animals, in agreement with the study described above [29]. In conjunction, these results suggested that OPG and RANKL were not directly associated with the lower level of bone neoformation observed in the hyperglycemic animals and that other metabolic pathways (Wnt/β-catenin, semaphorins, bone morphogenetic proteins, metalloproteinases) must be studied in an endeavor to elucidate the mechanism responsible for the reduction in alveolar bone neoformation associated with hyperglycemia.β

In the present study, the expression of osteocalcin and osteopontin in the alveoli of nonhyperglycemic and hyperglycemic rats was evaluated eight days after extraction. Osteocalcin and osteopontin are proteins associated with bone tissue formation, and in the present study, no significant differences were detected in the synthesis of these proteins in the post-extraction alveoli of hyper- and normoglycemic animals. Studies that have evaluated the involvement of osteocalcin and osteopontin in alveolar bone repair in diabetic animals are also scarce in the literature, which impede better discussion of the present study findings. Colombo et al. [30] described a reduction in the formation of mineralization tissue in animals and associated this result with increase in the presence of TNF-α, IL-1β, IL-6 in diabetic patients. More recently, Park and Kang [31] observed the beneficial effects of irradiation with 980 nm GaAlAs (*galium-aluminum-arsenide*) laser on the initial stages of post-extraction alveolar bone repair in diabetic and normoglycemic animals. Although the aims of the above-mentioned studies were not similar to those of the present study, the authors described the absence of differences in the osteocalcin and osteopontin levels in the alveoli of normo- and hyperglycemic animals, showing agreement with the data obtained in this investigation.

Relative to the markers studied in the present study, no reports were found in the literature in any study that had evaluated the effect of metformin on the expression of osteocalcin and osteopontin on bone repair. Although no effect of treatment with metformin was observed on the synthesis of OCN and OPN in the areas of alveolar bone repair of hyper- and normoglycemic animals, the *in vitro* study [31] demonstrated that metformin significantly inhibited the gene expression of RunX2, a fundamental transcription factor for the maturation of osteoblasts, together with the reduction in the expression of OCN, OPN and bone sialoprotein. The authors suggested that osteoblast differentiation is dependent on AMPK phosphorylation which could be inhibited by metformin.

The present study also showed that the normo- and hyperglycemic animals treated with metformin showed no difference in the OPG/RANKL proportion when compared with that of untreated animals. Results in disagreement were described [15], showing that metformin was capable of diminishing bone loss after the induction of periapical lesions in normoglycemic rats. Furthermore, the group of treated animals showed a reduction in the number of RANKL positive cells and increase in the number of OPG positive cells, in the time intervals of 14 and 28 days after induction of lesions, suggesting a bone-protective effect of this medication. However, it must be pointed out that the fructose treatment itself could affect intestinal calcium absorption particularly in chronic kidney disease or renal impairment that is very common in diabetic animals [32]. Therefore, the observed effects on bone tissue may also be a consequence of fructose-induced impairment of calcium absorption. In addition, differences in the conditions of tissue used for analysis (alveolus in repair and periapical lesion), and time of evaluation may explain the differences found. Finally, the absence of microCT evaluation should limited the better understanding of this process.

In an endeavor to understand the negative impact of hyperglycemia and treatment with metformin on alveolar bone repair, the present study evaluated the levels of SOX9, RunX2 and Osterix, transcription factors associated with osteoblast differentiation. Lower levels of expression of transcription factors RunX2 and SOX9 were detected in the initial stages of alveolar bone repair in hyperglycemic animals when compared with nonhyperglycemic rats. Up to now, no study has evaluated the expression of this transcription factor after extraction, relative to bone repair of fractures. Studies have demonstrated a delay in repair in diabetic animals, which is in agreement with the results obtained in the present study [33–35].

The cytokines in the supernatant of spleen cells isolated from nonhyperglycemic and hyperglycemic animals, either treated or not with metformin, in the absence or presence of ligature-inducted periodontitis were analyzed with the purpose of evaluating whether the presence of hyperglycemia either associated or not with periodontitis was capable of changing the systemic levels of cytokines associated with the inflammatory profile and of different subpopulations o auxiliary T lymphocytes (Th). ELISA analyses of the supernatants of *in vitro* unstimulated spleen cells with LPS showed that neither hyperglycemia nor the presence of ligature-induced periodontitis promoted increase in the systemic levels of the cytokines evaluated. Stimulation *in vitro* of the cells collected from animals in the different experimental groups was performed with the purpose of evaluating whether these animals had a higher capacity for responding to an infectious stimulus due to the pre-existent condition of hyperglycemia or ligature-induced periodontitis. It was possible to observe that after stimulus with LPS, there was an increase in the levels of TNF-α, IL-6, IL-4, IL-10 and IL-17. Hyperglycemia promoted an increase in the levels of IL-10 in comparison with those in nonhyperglycemic animals. The treatment of nonhyperglycemic animals with metformin was capable of mediating the increase in the levels of TNF-α, IL-10 and IL-17, while for the hyperglycemic animals increase in TNF-α and IL-17 was detected in the time intervals of 24 or 48 hours after stimulation with LPS. The ligature was capable of inducing the increase in the levels of TNF-α and IL-17 in both nonhyperglycemic and hyperglycemic animals. In conjunction, these data suggested that TNF-α and IL-17 are the cytokines most involved in the systemic response of experimental hyperglycemia and periodontitis, and consequently were the main targets of immunomodulation.

## 5. Conclusion

Taken together, the data of the present study revealed that the model and time of hyperglycemia used were incapable of mimicking the higher level of bone loss observed in the hyperglycemic animals and with ligature-induced periodontitis, however, immunohistochemical and histochemical analyses demonstrated a negative impact of hyperglycemia on alveolar bone tissue. Furthermore, the negative impact of hyperglycemia and/or treatment with metformin also could be noted in the initial stages of bone repair, via inhibition of transcription factors Runx2 and Sox9, involved in osteoblast differentiation. It was also shown that hyperglycemia, treatment with metformin and/or ligature-induced periodontitis promoted the increase in the *ex vivo* levels of TNF-α and IL-17, two cytokines involved in the inflammatory process and it’s possible to suggest that selective inhibition of these cytokines could be considered a possible immunomodulation to periodontitis associated with hyperglycemia.

## Supporting information

S1 Fig Study design. (JPEG)

S2 Fig Initial and final weight of animals belonging to nonhyperglycemic (NH) and hyperglycemic (H) groups. Dark bars represent the animals of Group NH, and white bars the animals of Group H. Letters represent differences between the initial and last body weight, evaluated by the Student’s-*t* test for dependent samples. Symbol represent significant difference among the groups relative to initial and last body weight, separately evaluated by the Student’s-*t* test for independent samples. No significant difference was observed relative to the initial body weight of the animals.
(JPEG)

S3 Fig Illustrative images of histological sections of alveoli of rats 8 days after extraction for Groups: Nonhyperglycemic (A), Nonhyperglycemic Treated (B), Hyperglycemic (C) and Hyperglycemic Treated (D). Arrows indicate the newly bone formation.
(JPEG)

S4 Fig Illustrative images of histological sections of alveoli of rats 8 days after extraction, after immunohistochemical reaction for OPG (A–D) and for RANKL (E- G) for Groups: Nonhyperglycemic (A and E), Nonhyperglycemic Treated (B and F), Hyperglycemic (C and G) and Hyperglycemic Treated (D and H). (JPEG)

S5 Fig Illustrative images of histological sections of alveoli of rats 8 days after extraction, after immunohistochemical reaction for OCN (A–D) and for OPN (E- G) for Groups: Control (A and E), Treated Control (B and F), Diabetic (C and G), Diabetic Treated (D and H). (JPEG)

S6 Fig Representative photomicrograph of mature osteoclasts expressing TRAP+ cells in furcation area of the mandibular first molar of hyperglycemic group. (A) histological section showing furcation area; (B) higher view showing a detail of the (A) figure.
(JPEG)
