# Supplementary figures and images for: Impact of hyperglycemia and treatment with metformin on ligature-induced bone loss, bone repair and expression of bone metabolism transcription factors
Source: PLoS One. 2020 Aug 25;15(8):e0237660. doi: 10.1371/journal.pone.0237660 (PMC7447028; doi:10.1371/journal.pone.0237660)

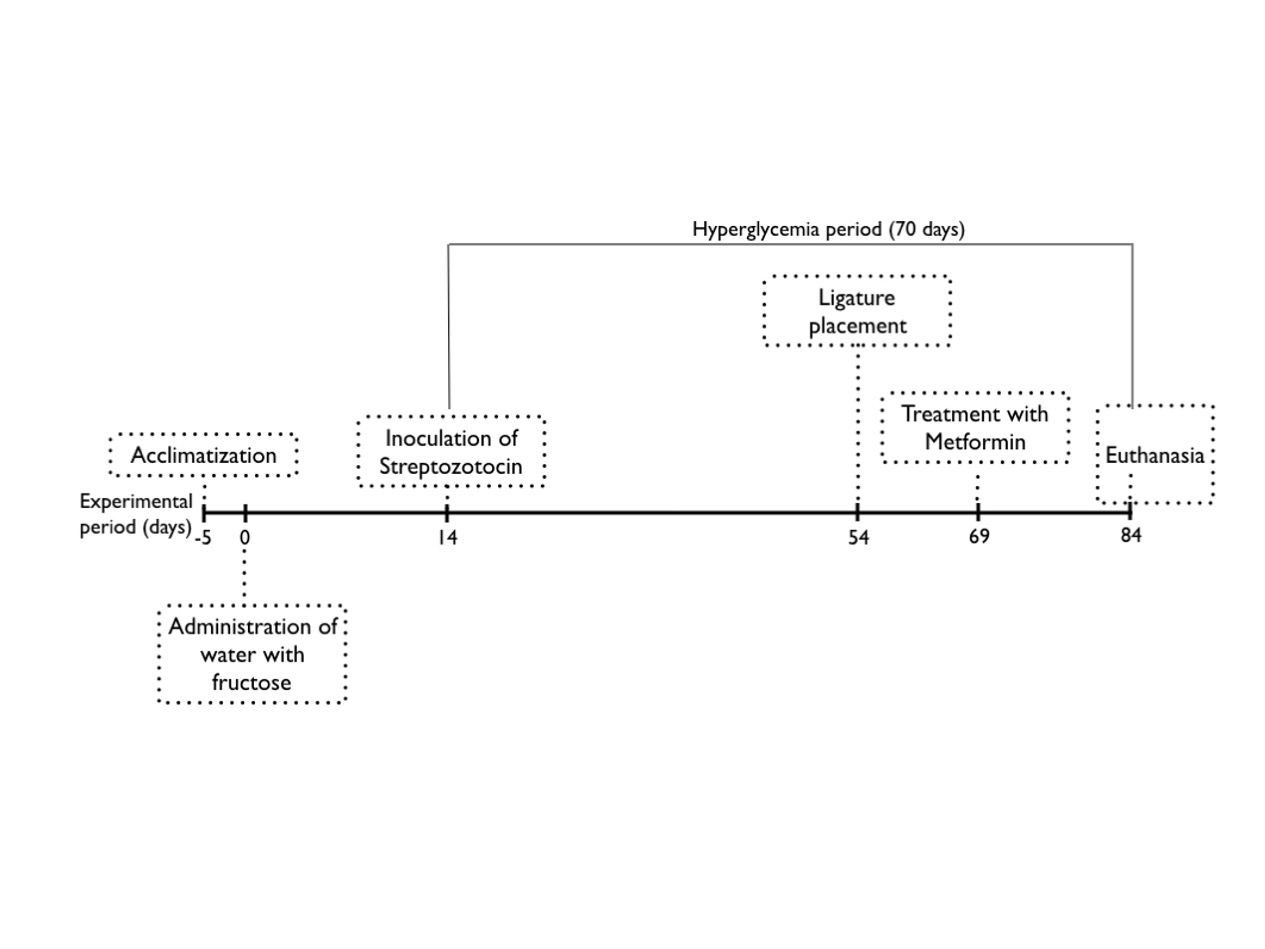

Supplement: S1 Fig — (JPEG) [file pone.0237660.s001.jpeg]

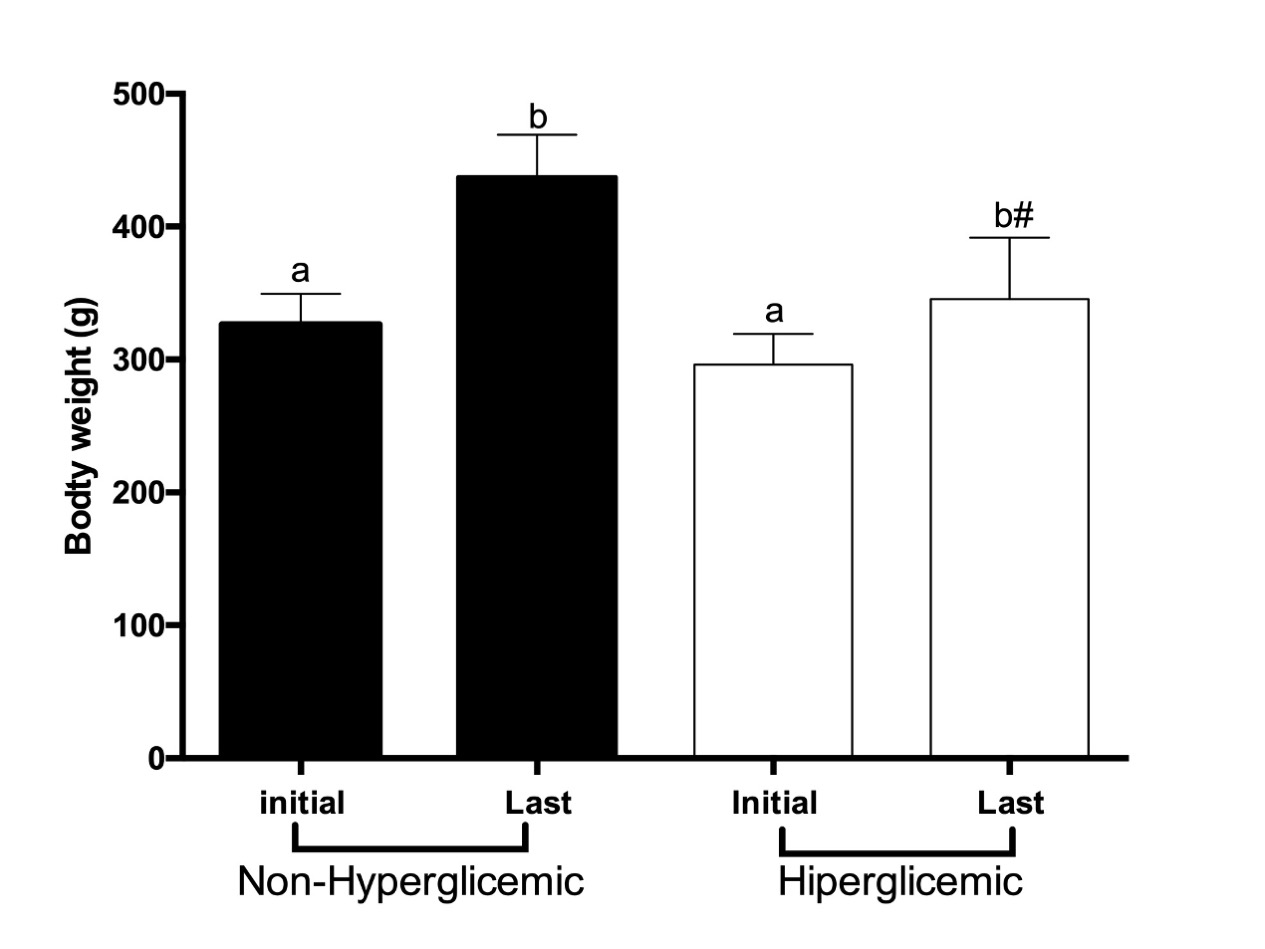

Supplement: S2 Fig — Dark bars represent the animals of Group NH, and white bars the animals of Group H. Letters represent differences between the initial and last body weight, evaluated by the Student’s-t test for dependent samples. Symbol represent significant difference among the groups relative to initial and last body weight, separately evaluated by the Student’s-t test for independent samples. No significant difference was observed relative to the initial body weight of the animals. (JPEG) [file pone.0237660.s002.jpeg]

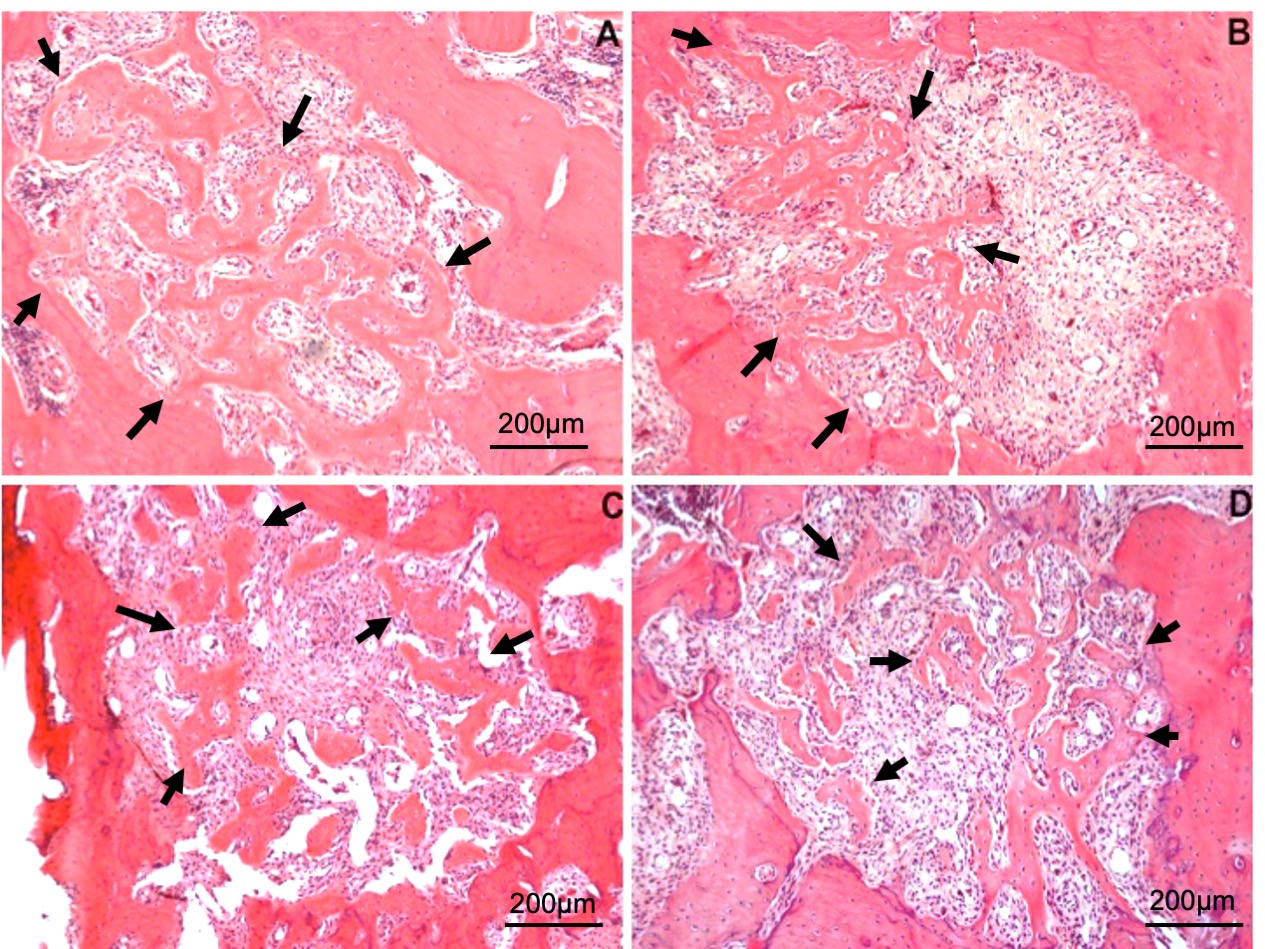

Supplement: S3 Fig — Arrows indicate the newly bone formation. (JPEG) [file pone.0237660.s003.jpeg]

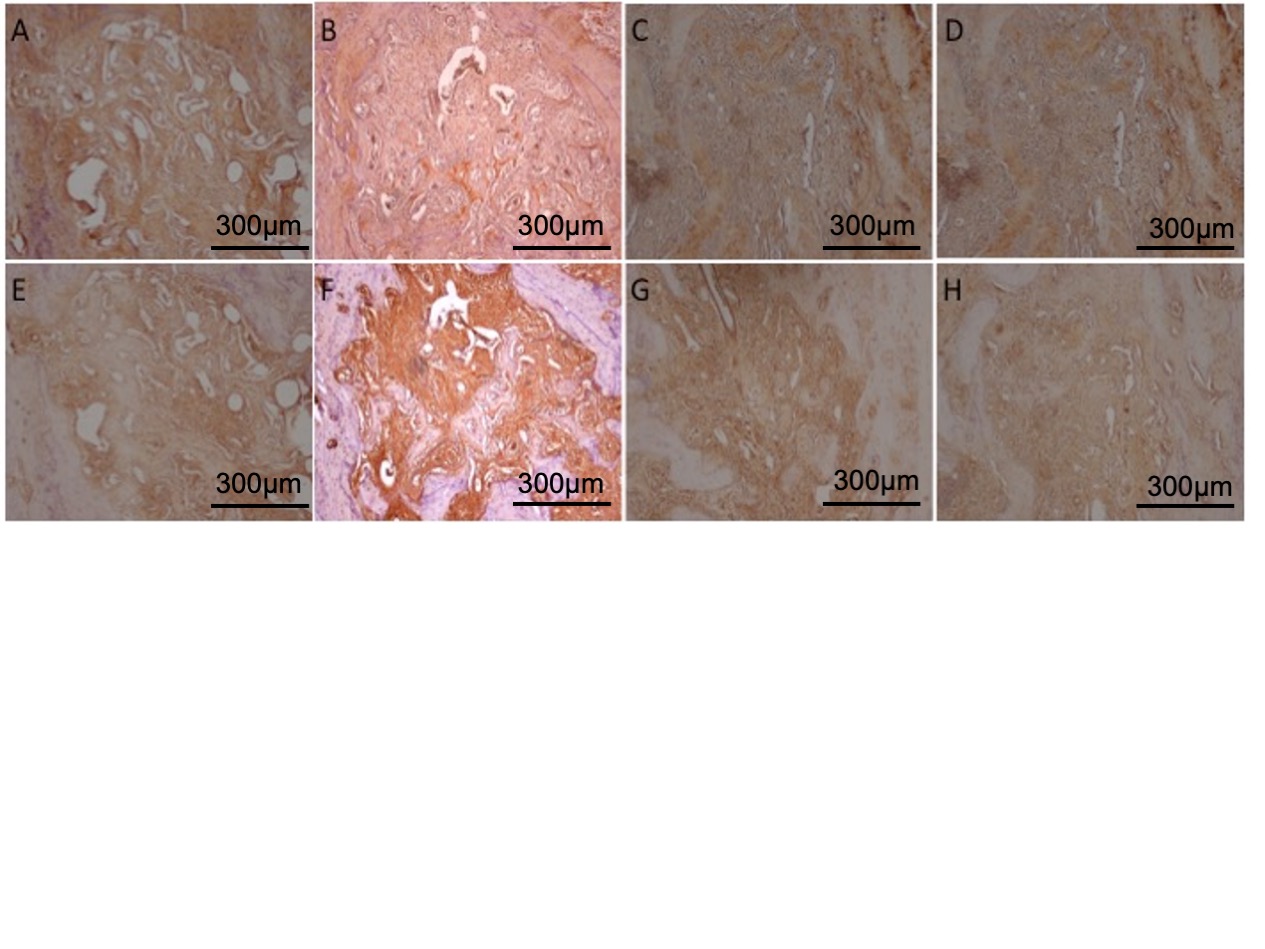

Supplement: S4 Fig — (JPEG) [file pone.0237660.s004.jpeg]

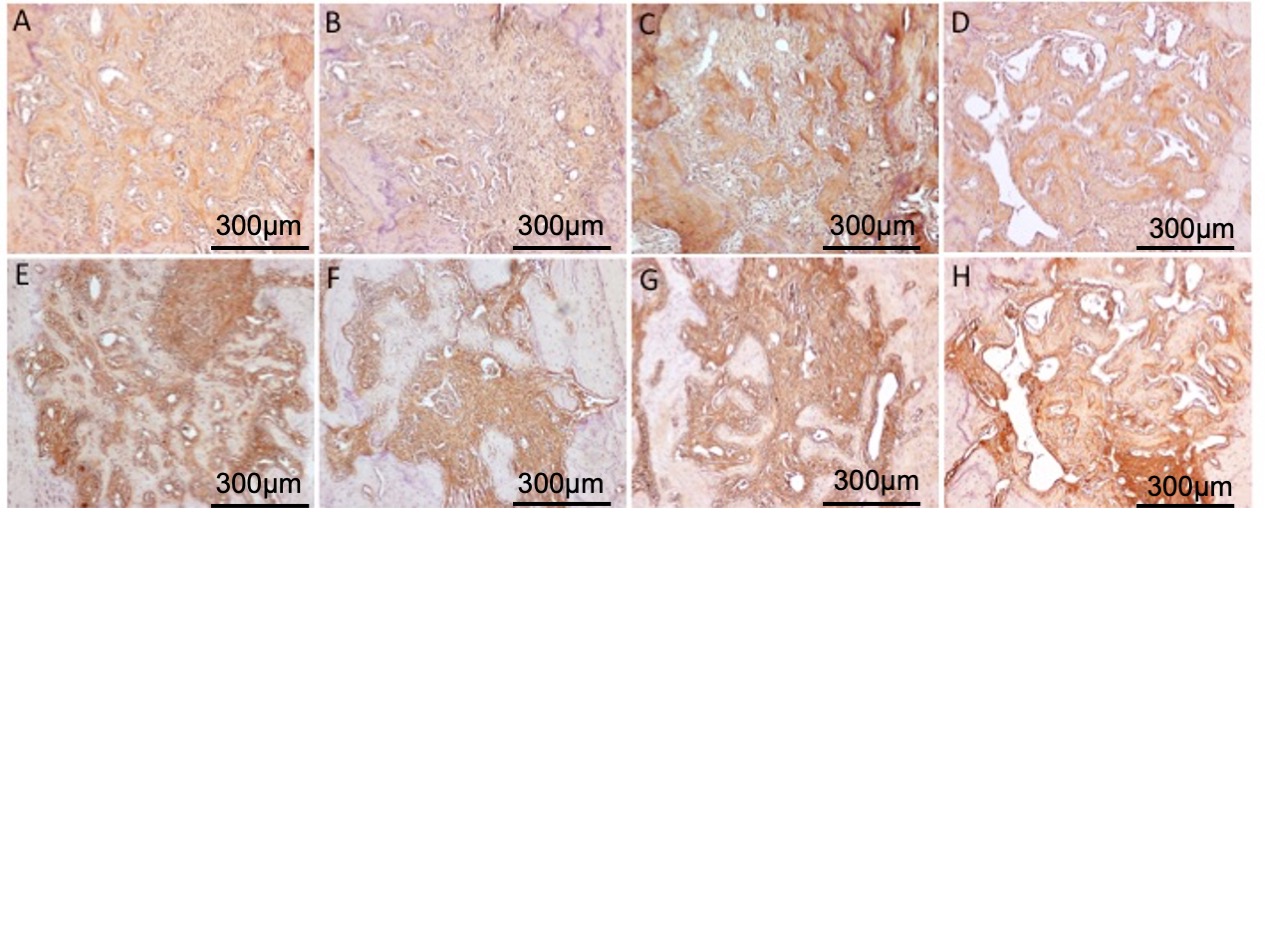

Supplement: S5 Fig — (JPEG) [file pone.0237660.s005.jpeg]

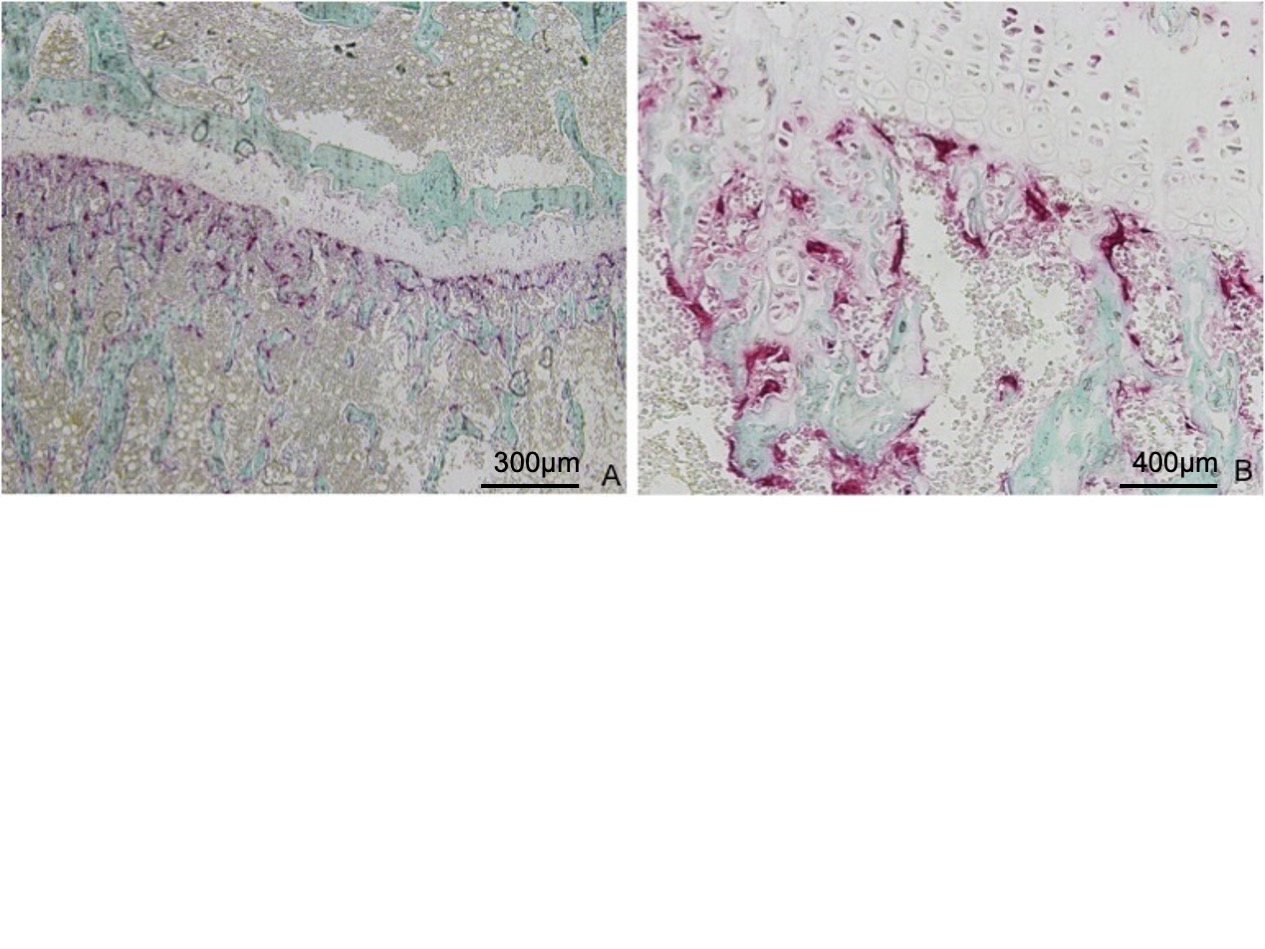

Supplement: S6 Fig — (A) histological section showing furcation area; (B) higher view showing a detail of the (A) figure. (JPEG) [file pone.0237660.s006.jpeg]
